# Upregulated CEMIP promotes intervertebral disc degeneration via AP‐1‐mediated change in chromatin accessibility

**DOI:** 10.1002/ctm2.70322

**Published:** 2025-05-21

**Authors:** Shibin Shu, Xin Zhang, Zhenhua Feng, Zhen Liu, Kaiyang Wang, Fengrui Li, Yating Wu, Bo Shi, Yong Qiu, Zezhang Zhu, Hongda Bao

**Affiliations:** ^1^ Division of Spine Surgery, Department of Orthopedic Surgery Nanjing Drum Tower Hospital, Affiliated Hospital of Medical School, Nanjing University Nanjing China

**Keywords:** CEMIP, chromatin accessibility, hyaluronic acid, intervertebral disc degeneration, nucleus pulposus

## Abstract

**Background:**

Intervertebral disc degeneration (IDD), a chronic and multifactorial skeletal disorder, is the primary cause of low back pain. It results in reduced disc height and nucleus pulposus hydration due to proteoglycan loss and nucleus pulposus cells (NPCs) dysfunction within a hypoxic microenvironment. Metabolic dysregulation initiates catabolic processes, leading to extracellular matrix (ECM) degradation and compromising disc biomechanical integrity. Emerging evidence highlights epigenetic modifications as pivotal in IDD, influencing NPC gene expression transcriptionally and post‐transcriptionally.

**Methods:**

In order to understand the epigenetic underpinnings of IDD, our study provided a comprehensive profile of chromatin accessibility changes in degenerated NPCs using Assay for Transposase‐Accessible Chromatin with high‐throughput sequencing (ATAC‐seq).

**Results:**

With motif enrichment analysis, we identified the activator protein‐1 (AP‐1) transcription factor critical in driving the chromatin accessibility changes during IDD. Integrative ATAC‐seq and transcriptional profiling revealed cell migration‐inducing protein (CEMIP) as a key biomarker and contributor to IDD, exhibiting marked upregulation in IDD. Furthermore, we demonstrated that the AP‐1 family, especially, c‐Fos, orchestrates the upregulation of CEMIP. Elevated CEMIP plasma levels correlated with clinical IDD severity, and CEMIP knockout mice demonstrated improved IDD.

**Conclusions:**

Mechanistically, CEMIP disrupted ECM homeostasis through its regulation of high molecular weight hyaluronic acid (HMW‐HA) degradation, and its contribution to fibrotic changes. Our findings highlight CEMIP's vital role in IDD and identify the AP‐1 family as a critical regulator of IDD, providing new potential therapeutic targets for novel IDD interventions.

**Key points:**

- Integrative ATAC‐seq and transcriptional profiling revealed CEMIPas a key biomarker and contributor to IDD, exhibiting marked upregulation in IDD.
- Further, we demonstrated that the AP‐1 family, especially, c‐Fos, orchestrates the upregulation of CEMIP.
- Elevated CEMIP plasma levels correlated with clinical IDD severity, and CEMIP knockout mice demonstrated improved IDD.
- Mechanistically, CEMIP disrupted extracellular matrix homeostasis through its regulation of high molecular weight hyaluronic acid degradation, and its contribution to fibrotic changes.
- Our findings offer new avenues for IDD treatment strategies, with the potential to alleviate the global burden of back pain.

## Graphical abstract

In degenerated NPCs, AP‐1 activation increased the accessibility of chromatin to transcriptionally upregulate CEMIP, while CEMIP disrupted ECM homeostasis through its regulation of HMW‐HA degradation, and its contribution to fibrotic changes within NP tissues. The AP‐1/CEMIP axis emerges as a novel target for the prevention and treatment of IDD.

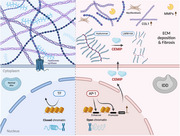

## 1 INTRODUCTION

Intervertebral disc degeneration (IDD), a chronic and multifactorial skeletal pathology, has been identified as the primary aetiology of low back pain. It manifests as a reduction in disc height and hydration of the nucleus pulposus, primarily due to proteoglycan loss and impairment of nucleus pulposus cells (NPCs) functions.^1^ Thus, IDD is thought to occur when the homeostatic balance of anabolic and catabolic metabolisms is lost and a predominantly catabolic and hypoxic microenvironment develops in the intervertebral disc (IVD), ultimately compromising the biomechanical integrity of the disc and adjacent spinal structures. Nevertheless, IDD, as a multifactorial disease, involves an interplay of environmental and genetic risk factors throughout an individual's lifespan, thereby necessitating further investigation to elucidate its pathogenesis.

Recent studies have highlighted the important role of epigenetic modifications in IDD pathogenesis, suggesting that both transcriptional and post‐transcriptional regulation of NPC gene expression may critically modulate disc degeneration. Genome‐wide DNA methylation profiling has presented different methylation patterns associated with IDD progression, revealing potential epigenetic biomarkers and pathways.^2^ For instance, enhanced methylation at specific loci, such as the promoter of the transient receptor potential ankyrin 1, has been involved in mitigating matrix degradation and inflammation in degenerated IVDs.^3^ This underscores the need to further reveal the epigenetic mechanisms that regulate IDD, particularly those governing gene expression via dynamic DNA regulatory elements such as enhancers and promoters.

Chromatin accessibility, which reflects the degree of chromatin compaction, plays a pivotal role in gene expression regulation by modulating the activity of cis‐regulatory elements where transcription factors (TFs) bind. Although comprising a minor fraction (about 3%) of the genome, these accessible regions host over 90% of TF binding sites, serving as the recruitment grounds for TFs to bind and thus influence gene expression. Alterations in chromatin accessibility are hallmarks of various diseases, including cancer,^4^ neurological disorders^5^ and osteoarthritis,^6^ highlighting their importance in disease pathogenesis. Additionally, TFs such as Pax1, Shh, Foxa1, T‐Brachyury and Sox5, 6, 9,^7^ play critical roles in NPC development and degeneration, with their activity potentially modulated by chromatin accessibility.

To advance our understanding of IDD, we employed the Assay for Transposase‐Accessible Chromatin with high‐throughput sequencing (ATAC‐seq), an advanced methodology for comprehensive profiling of genome‐wide chromatin accessibility, to delineate accessible and functionally active genomic loci. This technique leverages a hyperactive Tn5 transposase to concurrently fragment genomic DNA and integrate sequencing adaptors into accessible chromatin regions, facilitating targeted sequencing of these areas. The resultant dataset delineates an intricate map of DNA accessibility, pinpointing regulatory elements such as promoters and enhancers that are critical for modulating gene expression.^4^, ^5^ Our analysis revealed specific motifs that were markedly enriched within the accessible regions of our experimental samples, indicating their potential involvement in the regulatory network governing the biological process under investigation. By integrating ATAC‐seq data with motif enrichment analysis, we were able to formulate hypotheses concerning the mechanisms through which alterations in chromatin accessibility might induce changes in gene expression profiles.

Previous applications of ATAC‐seq have reported the chromatin landscapes in diseases such as osteoarthritis^6^ and rheumatoid arthritis,^8^ providing insights into disease‐specific chromatin dynamics. In this study, we applied ATAC‐seq to profile the chromatin accessibility in human degenerated NPCs. Integrating this with transcriptional data, we identified the TF activator protein‐1 (AP‐1) as a key player in NPCs degeneration. Moreover, we reveal cell migration‐inducing protein (CEMIP) as a novel AP‐1 target gene involved in NPCs degeneration. By revealing CEMIP's role in extracellular matrix (ECM) remodelling and fibrosis, we provide a fresh perspective on IDD pathophysiology and propose novel therapeutic targets. This integrative analysis of the NPCs transcriptional landscape casts light on the epigenetic complexities of IDD, spotlighting the AP‐1‐mediated upregulation of CEMIP as a significant pathological biomarker.

## 2 METHODS

### 2.1 Patient recruitment and sample collection

This study was conducted in accordance with the ethical guidelines approved by the Ethics Committee of Nanjing Drum Tower Hospital, Affiliated Hospital of Nanjing University Medical School (approval no. 2023‐506‐01). Informed consent was obtained from all participants. Normal lumbar nucleus pulposus (NP) tissues and peripheral blood plasma were collected from 15 patients undergoing surgery due to congenital scoliosis or thoracolumbar fracture at the Department of Spine Surgery, Nanjing Drum Tower Hospital (seven males and eight females; age 8–52 years, mean age 25.3 years). Degenerative lumbar NP tissues and plasma were obtained from 15 patients undergoing spinal fusion surgery for degenerative disc disease (six males and nine females; age 39–76 years, mean age 57.7 years) (Table S6). The human NP samples were classified according to their degenerative grades. Five grades were assigned to T2‐weighted magnetic resonance imaging (MRI) images, representing a progression from normal disc to severe disc degeneration, where grade I corresponded to no degeneration while grade V represented the most severe degeneration.^9^ Scoring was implemented for easy evaluation, to grade I, one score was given, while for grade V, five scores were given. Higher scores indicated a greater severity of IDD. The cumulative score for disc degeneration from L1‒L2 to L5‒S1 was calculated and defined as the lumbar disc score.^10^

### 2.2 Isolation and culture of NP cells

Human NP samples were processed to establish primary cell cultures. After cutting into pieces, the human NP tissues were digested in  0.2% type II collagenase (Gibco) for 4 h. The digested tissue was filtered, washed with phosphate‐buffered saline (PBS), and centrifuged to isolate cells. The isolated NP cells were cultured in Dulbecco's modified Eagle medium—low glucose (1 g/L; Gibco) supplemented with 10% foetal bovine serum (Gibco) and 1% penicillin‒streptomycin (Gibco). The culture medium was replaced every 3 days. NP cells were stimulated with 10 ng/mL interleukin‐1β (IL‐1β) (Sigma) to establish a degeneration model.^11^, ^12^

### 2.3 siRNA and plasmid transfection

NP cells were transfected with siRNAs targeting CEMIP, c‐Fos, Fra‐1, ATF3 or negative control (NC) siRNA (Hippobio) using Lipofectamine 3000 (ThermoFisher Scientific), following the manufacturer's protocols. The most effective siRNA sequences were utilised based on preliminary studies (Figure S3 and Table S7). For plasmid transfections, NP cells were transfected with either NC plasmid or CEMIP plasmid (Syngentech) following the manufacturer's instructions.

### 2.4 ATAC‐seq and analysis

Fifty thousand NP cells were isolated and processed for ATAC‐seq following established protocols.^13^, ^14^ Briefly, nuclei were extracted for transposition reactions, and DNA was purified and amplified to construct libraries. Libraries were quantified and sequenced using BGI‐seq 500 platform (BGI Genomics Co., Ltd.). We referred to cisDynet^15^ computational pipeline to perform the ATAC‐seq data analysis. Briefly, we first removed the potential sequencing adapters with Trimmomatic^16^ with the default parameters. We next aligned the reads to the human genome (hg38) with the mem function from the BWA^17^ software. Only uniquely mapped paired reads were sorted using SAMtools and kept for subsequent analysis. We then used the MACS2 to perform peak calling, with the following parameters: –g 1.3e+8, –nomodel –extsize 150 –shift 75. We used irreproducible discovery rate (IDR) to filter the set of reliable peeks between replicates to do downstream analyses. Bigwig files were generated by bamCoverage function from deepTools software with the default parameters. We use Diffbind for differential peak identification. For differential peaks, we used FIMO and Homer for motif enrichment analysis, respectively. We used TOBIAS for footprint identification.

### 2.5 RNA sequencing and analysis

Total RNA was extracted from NP cells using Trizol, with mRNA enrichment, fragmentation and reverse transcription into cDNA. Sequencing was performed on the BGI‐seq 500 platform (BGI Genomics Co., Ltd.). We used RSEM^18^ to quantify RNA sequencing (RNA‐seq). Differentially expressed genes (DEGs) were identified (DESeq2 package; |fold change| ≥ 2, false discovery rate < .05) and subjected to Gene Ontology (GO) enrichment analysis and gene set enrichment analysis (GSEA).

### 2.6 Dual‐luciferase reporter assay

Human embryonic kidney 293T cells were plated in 48‐well plates and transiently co‐transfected with luciferase reporter constructs and CEMIP expression vectors, including PGL3, PGL3‐Fos, PGL3‐Jun, PGL3‐Fra‐1 and PGL3‐Atf‐3. Forty‐eight hours post‐transfection, luciferase activity was assayed using the dual‐luciferase reporter assay system.

### 2.7 Western blot analysis

Proteins from NP cells and tissues were extracted with radioimmunoprecipitation assay buffer. Proteins were then resolved on SDS‒polyacrylamide gels and transferred to polyvinylidene fluoride membranes (Bio‐Rad). Membranes were blocked with 5% skimmed milk at room temperature for 1 h and incubated with primary antibodies at 4°C overnight. After incubation with secondary antibodies for 1 h, protein bands were visualised using the ChemiDoc XRS+ Imaging System (Tanon). This protocol was repeated three times for reproducibility. Primary antibody details can be found in Table S8.

### 2.8 Quantitative real‐time PCR

Total RNA was isolated from NP cells utilising TRIzol reagent (ThermoFisher), and reverse transcribed to complementary DNA (cDNA) using reverse transcription kits (Vazyme Biotech). Quantitative PCR (qPCR) analyses were performed on a LightCycler 480 II system (Roche Molecular Biochemicals) with specific primers listed in Table S9.

### 2.9 Immunohistochemical and immunofluorescent staining

Tissue sections were processed for immunohistochemical (IHC) staining as per the provided protocol. After overnight incubation with primary antibodies at 4°C, sections were incubated with horseradish peroxidase (HRP)‐conjugated secondary antibodies at 37°C for 1 h. While for immunofluorescent (IF) staining, fluorescein isothiocyanate (FITC)‐ or tetramethyl rhodamine isothiocyanate (TRITC)‐conjugated secondary antibodies were used for sections under a dark condition for 1 h. Stained sections were visualised and imaged using a fluorescence microscope (Zeiss). Details of the primary antibodies are included in Table S8.

### 2.10 Cell immunofluorescent staining

Cells were fixed in 4% paraformaldehyde and permeabilised with  .1% Triton X‐100. Blocking was performed using 5% bovine serum albumin for 1 h at 37°C. Incubation with primary antibodies was carried out overnight at 4°C, details of which are listed in Table S8. Afterward, cells were exposed to FITC‐ or TRITC‐conjugated secondary antibodies and counterstained with DAPI. Fluorescence microscopy (Zeiss) was employed for visualisation.

### 2.11 Enzyme‐linked immunosorbent assay

Conditioned media from treated NP cells were preserved at −80°C. Human plasma samples were procured from collected patient peripheral blood. CEMIP levels in cell supernatant and plasma were quantified using a human CEMIP ELISA Kit (Mlbio; ml002038), following the manufacturer's protocol. Similarly, high molecular weight hyaluronan (HMW‐HA) concentrations were measured with a HMW‐HA ELISA Kit (Mlbio; ml001898S).

### 2.12 Cell proliferation assay

NP cells were cultured in 96‐well plates. Cell proliferation was assessed with a Cell Counting Kit‐8 (Solarbio), adhering to the manufacturer's guidance. Absorbance at 450 nm was measured using microplate readers (Thermo Scientific).

### 2.13 Generation of CEMIP‒/‒ mice by CRISPR/Cas9 technology

CRISPR/Cas9‐mediated CEMIP knockout mice (Cas9‐KO, strain no. T010322) were acquired from GemPharmatech. The CEMIP gene has five transcripts. According to the structure of CEMIP gene, exon3‒exon5 of CEMIP‐201 (ENSMUST00000064174.11) transcript is recommended as the knockout (KO) region. The region contains 523 bp coding sequence. Knock out the region will result in disruption of protein function. Heterozygous CEMIP± mice were bred with wild‐type (WT) C57BL/6J mice to produce homozygous CEMIP‒/‒ KO progeny (Figure S4). Mice were maintained at 21°C and 55% relative humidity with a 12‐h light/dark cycle. All animal experiment protocols were authorised by the Animal Center of Nanjing Drum Tower Hospital, the affiliated Hospital of Nanjing University Medical School (approval no. DWSY‐22088214).

### 2.14 Coccygeal IVDs needle stab IDD model

A needle stab model was established to induce IDD in 12‐week‐old homozygous CEMIP KO mice as previously described.^19^ A 31‐G needle was inserted 1.5 mm into the Co6‐7 IVD of mice under a small sagittal incision extending from Co6 to Co8. The needle was rotated 180° axially and held for 10 s. The Co7‒8 disc was left intact and served as a control. Six weeks post‐procedure, caudal IVDs were evaluated using micro‐computed tomography (µCT) and MRI, and tissues were preserved in formalin for histological analysis.

### 2.15 Histological and microscopic analyses

NP tissues from both murine (coccygeal segments Co6‒7 and Co7‒8) and human samples were fixed in 4% (v/v) paraformaldehyde for 24 h and subsequently decalcified in 10% (v/v) ethylenediaminetetraacetic acid (EDTA) for 2 months. Tissues were then embedded in paraffin and sectioned sagittally at a thickness of 5 µm. Sections underwent staining with haematoxylin and eosin, Safranin O‐fast green and Masson's trichrome. A standard histological grading system was employed to evaluate the samples.^20^

### 2.16 Micro‐computed tomography

The µCT system (mCT80; Scanco Medical AG) was preferred over conventional X‐ray for the quantification of the intervertebral disc height index (DHI), offering a more direct assessment of disc height alterations. Three‐dimensional reconstructions were obtained using Scanco Medical software. DHI was calculated following a previously established method.^21^

### 2.17 Magnetic resonance imaging

Animal subjects were imaged using a 9.4 T MRI system (BioSpec 94/20 USR, Bruker). T2‐weighted images were specifically utilised to assess disc hydration status. Disc degeneration was graded applying the Pfirrmann grading system, as previously described.^9^

### 2.18 Detection of high molecular weight HA by high‐performance liquid chromatography

Supernatants from human NP cell cultures were collected post‐treatment and stored at ‒80°C until analysis. The samples were concentrated 10‐fold using centrifugal filter units (VS2052, Sartorius), which selectively retained substances above 100 kDa (Figure S5). After centrifugation to remove insoluble materials, the samples were lyophilised. The resuspended precipitates were subjected to high‐performance liquid chromatography (HPLC) using a DIONEX Ultimate 3000 system (Thermo Fisher) equipped with a C18 column (2.1 mm ×100 mm, 1.8 µm) at 35°C. These precipitates above were dissolved in the mobile phase (400 µL). The samples were filtered through a  .22 µm membrane prior to a 10 µL injection with an Altex 210 injector. A  .05 M Na_2_SO_4_ solution served as the mobile phase at a flow rate of 1 mL/min, with the sample chamber maintained at 4°C.

### 2.19 Statistical analysis

Data are presented as mean ± standard deviation from at least three independent experiments. Two‐group comparisons were analysed using unpaired Student's *t*‐tests. For multiple group comparisons, one‐way analysis of variance was utilised, followed by post hoc test for multiple comparisons. The correlation statistical analyses between variables were performed using the Spearman two‐tailed correlation test. All statistical procedures were executed using GraphPad Prism 9 (GraphPad Software Inc.), with a *p*‐value of less than  .05 considered statistically significant.

## 3 RESULTS

### 3.1 Chromatin accessibility landscape undergoes substantial changes during IDD

**FIGURE 1:**
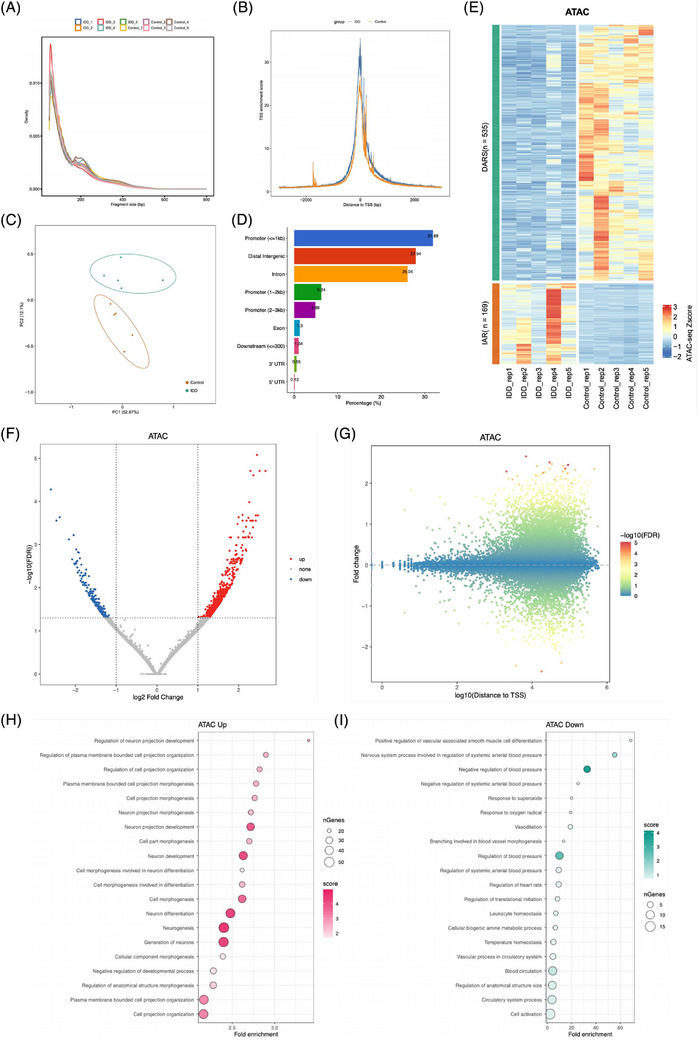
Genome‐wide landscape changes of chromatin accessibility for human degenerated nucleus pulposus cells (NPCs). (A) Assay for Transposase‐Accessible Chromatin with high‐throughput sequencing (ATAC‐seq) sequencing fragment length distribution for normal (*n* = 5) and degenerated (*n* = 5) NPCs libraries. (B) ATAC‐seq signals were abundantly enriched at the proximal transcription start sites (TSSs). (C) Principal component analysis (PCA) analysis showing the separate clustering of different samples and the clustering of each replicate of the same condition together. (D) Distribution categories of all chromatin accessible regions in the genome. UTR, untranslated region. (E) Heatmap of hierarchically clustered ATAC‐seq signals showing the accessibility remodelling of intervertebral disc degeneration (IDD)‐related chromatin regions with increased accessibility (IARs) and regions with decreased accessibility (DARs) in NPCs. (F) Volcano plot comparing chromatin accessibility peaks in normal NPCs to degenerated NPCs. Blue points are more accessible in normal NPCs and red points are more accessible in degenerated NPCs (fold change ≥ 2, *p*‐value adjusted ≤  .05). (G) Scatter diagram showing alterations of ATAC‐seq signals between normal and degenerated NPCs in all peaks and with different distances to TSSs. Most of the regions that changed markedly in IDD are far from their TSSs. FDR, false discovery rate. (H and I) Gene Ontology (GO) enrichment of genes near IARs and DARs in degenerated NPCs.

To determine the chromatin accessibility changes of NPCs that occur following IDD, we performed the ATAC‐seq on degenerated NPCs isolated from five IDD patients and normal NPCs isolated from five human normal discs (derived from scoliosis patients). Quality control of the sequencing data from the degenerated NPCs and normal NPCs showed that most of the inserted fragments were small fragments containing <200 bp, representing open chromatin regions without nucleosome occupation and with mono‐ and double‐nucleosome distribution patterns (Figure 1A). Additionally, ATAC‐seq signals were abundantly enriched at the proximal transcription initiation sites (TSS) (Figure 1B). Principal component analysis revealed that the degenerated NPCs and normal NPCs were clustered by group and significantly distinguished from each other, reflecting profound changes in chromatin organisation associated with IDD (Figure 1C).

A thorough mapping of accessible chromatin regions during NPC degeneration highlighted a predominance in promoters and intergenic and intronic regions, with enhancers representing a significant proportion (Figure 1D). In degenerated NPCs, comparative analysis with normal NPCs identified 169 genomic regions with increased accessibility (IARs) and 535 regions with decreased accessibility (DARs) (fold change ≥ 2, Figure 1E‒G). GO analysis of genes near IARs in degenerated NPCs revealed enrichment in biological processes such as ‘cell morphogenesis’ and ‘cell adhesion’, alongside ‘proteoglycan metabolic process’ and ‘positive regulation of hyaluronan biosynthetic process’ (Figure 1H and Table S1). This aligns with previous findings that RNA methylation and histone modifications modulate IVD degeneration through ECM homeostasis.^22^ Conversely, genes near DARs did not show significant enrichment in IDD‐related processes (Figure 1I and Table S1). Altogether, our findings reveal an altered chromatin landscape in NPCs during degeneration, suggesting that increased chromatin accessibility is a prerequisite for the activation of multiple genes and signalling pathways in NPC degeneration.

### 3.2 Motif analysis suggests a role for AP‐1 transcription factors in regulating the NPCs degeneration

**FIGURE 2:**
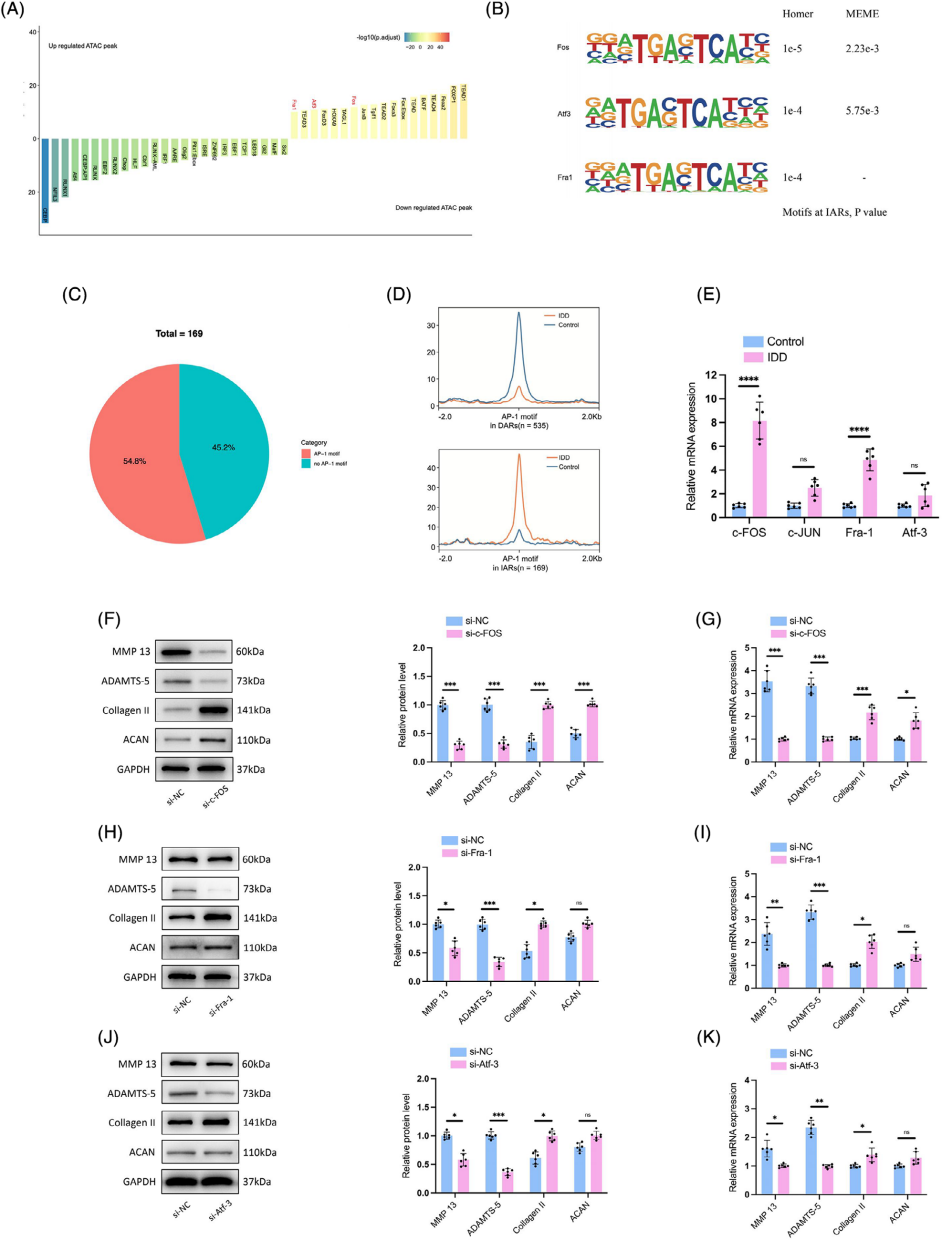
Assay for Transposase‐Accessible Chromatin with high‐throughput sequencing (ATAC‐seq) analysis reveals a pivotal role for activator protein‐1 (AP‐1) transcription factors in regulating chromatin dynamics in degenerated nucleus pulposus cells (NPCs). Motif analysis results using Homer (A) and MEME (B) algorithms for transcription factors at regions with increased accessibility (IARs) and regions with decreased accessibility (DARs). (C) Analysis of motif enrichment in IARs in degenerated NPCs reveals the significant enrichment of AP‐1 motifs, found in 54.8% of regions with increased chromatin accessibility. (D) Comparisons of ATAC‐seq signals of AP‐1 motif at IARs and DARs. (E) qRT‐PCR analyses of c‐Fos, c‐Jun, Fra‐1 and ATF3 in degenerative and normal NPCs. *n* = 6. (F) Western blotting analyses showing protein levels of matrix metalloproteinase‐13 (MMP‐13), a disintegrin and metalloproteinase with thrombospondin motifs subtype 5 (ADAMTS‐5), Collagen II and Aggrecan (ACAN) in NPCs after c‐Fos knockdown. *n* = 6. (G) RT‐qPCR results showing mRNA levels of MMP‐13, ADAMTS‐5, Collagen II and ACAN in NPCs after c‐Fos knockdown. *n* = 6. (H) Western blotting analyses showing protein levels of MMP‐13, ADAMTS‐5, Collagen II and ACAN in NPCs after Fra‐1 knockdown. *n* = 6. (I) RT‐qPCR results showing mRNA levels of MMP‐13, ADAMTS‐5, Collagen II and ACAN in NPCs after Fra‐1 knockdown. *n* = 6. (J) Western blotting analyses showing protein levels of MMP‐13, ADAMTS‐5, Collagen II, and ACAN in NPCs after ATF3 knockdown. *n* = 6. (I) RT‐qPCR results showing mRNA levels of MMP‐13, ADAMTS‐5, Collagen II and ACAN in NPCs after ATF3 knockdown. *n* = 6. NS: no statistical significance, ^*^ *p* < .05, ^**^ *p* < .01, ^***^ *p* < .001, ^****^ *p* < .0001.

To identify TFs driving chromatin accessibility changes in NPCs during degeneration, we applied HOMER (Figure 2A) and MEME algorithms (Figure 2B) for motif enrichment analysis within both IARs and DARs. This dual approach consistently identified the AP‐1 TF family as prominently enriched in IARs, with AP‐1 motifs present in over half (54.8%) of these regions in degenerated NPCs, suggesting a vital role in the degenerative process (Figure 2A‒C). Also, ATAC‐seq signals at AP‐1 motifs decreased in DARs in degenerated NPCs, an opposite effect to that in IARs (Figure 2D).

AP‐1, comprised of immediate‐early response genes including JUN, FOS (c‐Fos, FosB, Fra‐1 and Fra‐2), ATF and MAF, is known to respond to various extracellular stressors.^23^ Given our motif enrichment analysis revealed c‐Fos, Fra‐1 and ATF3 as significantly enriched (Figure 2A), we sought to validate whether AP‐1 TFs function during NPCs degeneration and which subunits of AP‐1 are involved during IDD. The qRT‐PCR analysis confirmed elevated c‐Fos and Fra‐1 expression in NPCs derived from IDD patients, while ATF3 and c‐JUN levels were unchanged when compared to normal NPCs (Figure 2E). Subsequent knockdown experiments of c‐Fos, Fra‐1 or ATF3 showed an increase in anabolic ECM proteins, Aggrecan (ACAN) and Collagen II, and a decrease in catabolic enzymes matrix metalloproteinase‐13 (MMP‐13) and a disintegrin and metalloproteinase with thrombospondin motifs subtype 5 (ADAMTS‐5), with the most notable effects observed following c‐Fos suppression (Figure 2F‒K). These findings align with prior research indicating AP‐1's role in regulating inflammatory responses via IL‐17 in human NPCs.^24^ Taken together, the data highlight the AP‐1 family, particularly c‐Fos, as key regulators of NPCs degeneration, orchestrating the degenerative process through chromatin accessibility remodelling in IARs, and potentially influencing both anabolic and catabolic pathways within the ECM.

### 3.3 Integrative transcriptomics and epigenomics analysis reveal that CEMIP contributes to NPCs degeneration regulated by c‐Fos

**FIGURE 3:**
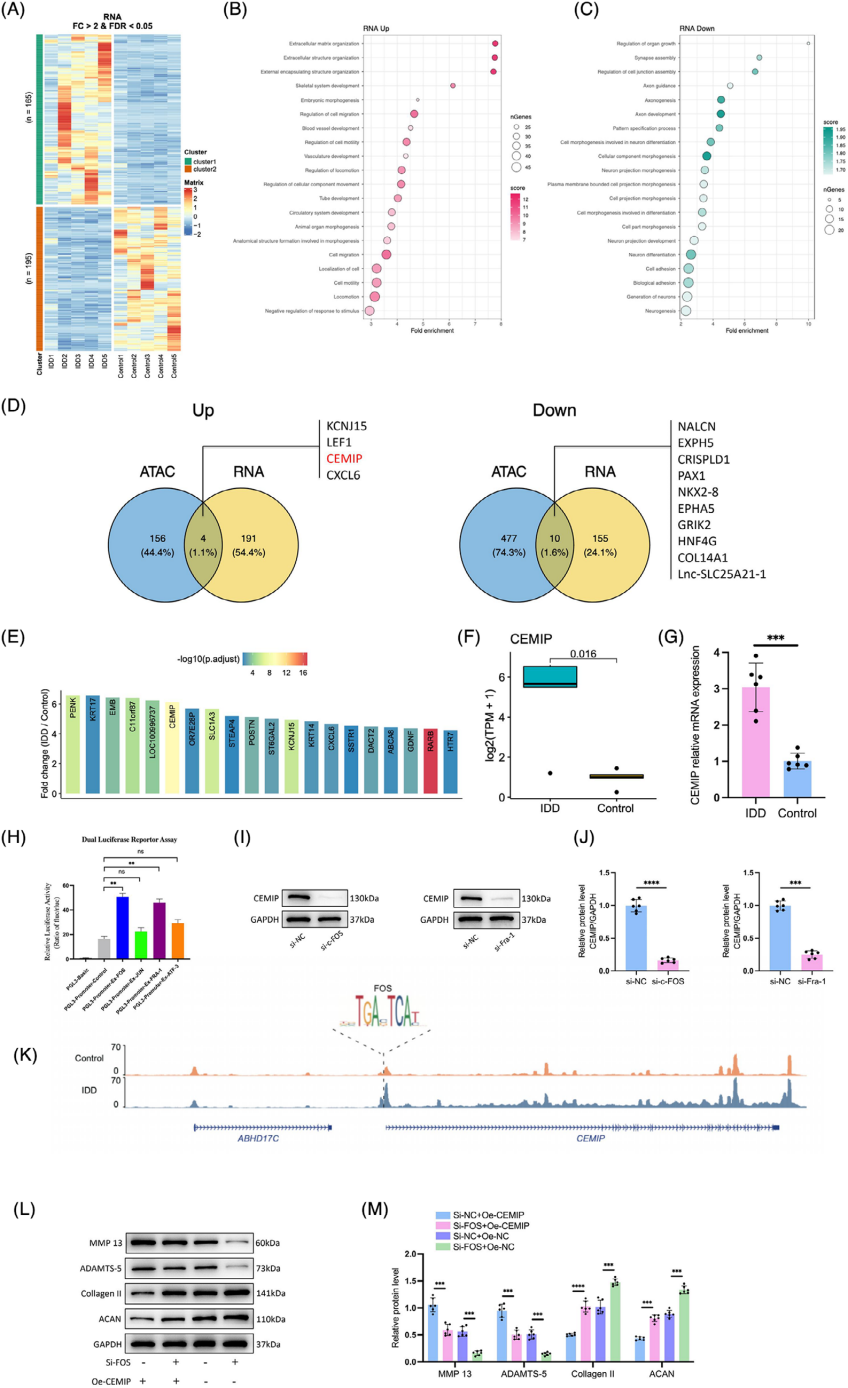
c‐Fos is the upstream actor that drives cell migration‐inducing protein (CEMIP) transcription in degenerated nucleus pulposus cells (NPCs). (A) Heatmap showing the differential expressed genes identified by DEseq2. Red and green colours represent high and low expression levels, respectively. (B and C) Gene Ontology (GO) enrichment analysis of differentially expressed genes (DEGs) identified by RNA sequencing (RNA‐seq). Only BP terms were shown. (D) Overlapped DEGs identified by Assay for Transposase‐Accessible Chromatin with high‐throughput sequencing (ATAC‐seq) and RNA‐seq. (E) Bar chart representing the fold change in the top targeted DEGs. Bar corresponding to CEMIP shows an upregulation in the degenerated NPCs, with a moderate level of statistical significance indicated by its yellow colour. Expression of CEMIP was compared between degenerated and normal NPCs quantified by RNA‐seq (F) and qRT‐PCR (G). (H) Transcriptional activity of CEMIP in 293T cells transfected by PGL3, PGL3‐Fos, PGL3‐Jun, PGL3‐Fra‐1 or PGL3‐Atf‐3. *n* = 6. (I and J) Degenerated NPCs were transfected with non‐target control siRNA (si‐NC), specific siRNAs targeting c‐Fos (si‐c‐FOS) or specific siRNAs targeting Fra‐1 (si‐Fra‐1), the expression of CEMIP was compared using Western blot analyses. *n* = 6. (K) motif footprint analysis showed c‐Fos motif was enriched in the significant peaks in the promoter region of CEMIP. (L and M) Rescue of the effect of c‐Fos knockdown by CEMIP overexpression. CEMIP‐overexpressing cells (Oe‐CEMIP) and control NPC cells were transfected with si‐NC or si‐c‐FOS, and the protein levels of matrix metalloproteinase‐13 (MMP‐13), a disintegrin and metalloproteinase with thrombospondin motifs subtype 5 (ADAMTS‐5), Collagen II and Aggrecan (ACAN) were traced by Western blot analyses. *n* = 6. NS: no statistical significance, ^**^ *p* < .01, ^***^ *p* < .001, ^****^ *p* < .0001.

High‐throughput RNA‐seq was performed to evaluate the transcriptome of degenerated NPCs. A total of 360 DEGs were revealed in degenerated NPCs compared to normal NPCs, including 195 upregulated and 165 downregulated genes (Figure 3A). GO enrichment analysis showed these upregulated genes were predominantly associated with ECM processes like ‘extracellular matrix organisation’, ‘extracellular structure organisation’, ‘external encapsulating structure organisation’ and ‘cell migration’, while downregulated genes were linked to ‘cellular component morphogenesis’, ‘cell adhesion’ and ‘biological adhesion’ (Figure 3B,C and Table S2). This GO analysis underscores the involvement of ECM remodelling in NPC degeneration.

We identified target genes according to the nearest TSS assignment principle for the identified differential open chromatin regions, respectively, and then overlapped with the RNA‐seq differential genes (Figure 3D). This integrative approach highlighted the CEMIP as a significantly upregulated gene involved in NPCs degeneration (Figure 3D‒F). qRT‐PCR validation confirmed elevated CEMIP expression in IDD‐derived NPCs, supporting its potential as an important molecular in degeneration (Figure 3G).

Given the established role of the AP‐1 family in NPC degeneration, we investigated their influence on the CEMIP promoter using a luciferase reporter assay. Both c‐Fos and Fra‐1 notably enhanced the luciferase activity of CEMIP promoters, indicating increased transcriptional activation, while ATF‐3 and c‐Jun had less luciferase activity (Figure 3H). The downregulation of CEMIP following c‐Fos or Fra‐1 knockdown validated their regulatory role of CEMIP (Figure 3I,J). Moreover, we found a differential open chromatin region in the CEMIP promoter region with a c‐FOS footprint by analysing the ATAC‐seq data (Figure 3K). The above data suggested that c‐Fos could be recruited at TCF‐binding sites located on the CEMIP promoter.

Rescue experiments further validated the regulatory relationship between CEMIP and AP‐1 subunits. Silencing c‐Fos via siRNA upregulated key ECM components such as ACAN and Collagen II and downregulated MMP‐13 and ADAMTS‐5, suggesting a regulatory role for c‐Fos in ECM metabolism (Figure 3L,M). Conversely, the influences of knocking down c‐Fos were restored with overexpressing CEMIP (Figure 3L,M). Similar rescue experiments of CEMIP and Fra‐1 also validated this regulatory axis between the CEMIP and the AP‐1 family (Figure S1). These analyses indicate that CEMIP may function downstream or in parallel with the AP‐1 family, significantly contributing to the signalling pathways that regulate ECM homeostasis and NPC degeneration.

### 3.4 CEMIP is highly expressed in the nucleus pulposus and the blood circulation of IDD patients

**FIGURE 4:**
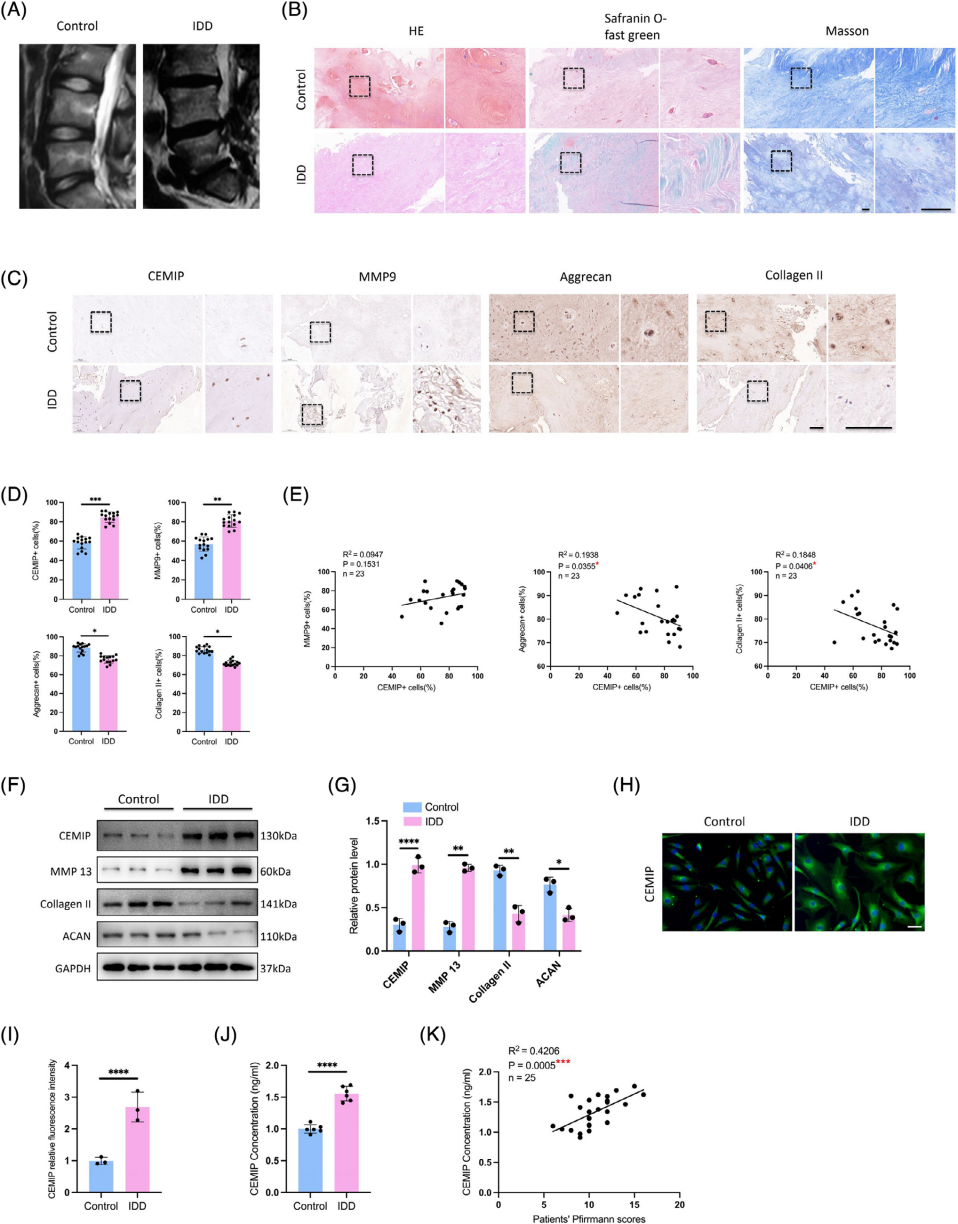
Cell migration‐inducing protein (CEMIP) is highly expressed in the nucleus pulposus and the blood circulation of intervertebral disc degeneration (IDD) patients. (A) T2‐weighted magnetic resonance imaging (MRI) of degenerative and normal intervertebral discs in patients. (B) Haematoxylin and eosin (HE) staining, Safranin O‐fast green and Masson staining of degenerative and normal nucleus pulposus tissues. Scale bar: 50 µm. (C and D) Immunohistochemistry (IHC) staining of CEMIP, matrix metalloproteinase‐9 (MMP‐9), Aggrecan (ACAN) and Collagen II in human NP samples. Scale bar: 100 µm. *n* = 15. (E) Correlation between CEMIP+ cells (%) and MMP‐9+ cells (%), ACAN+ cells (%) and Collagen II+ cells (%) of IHC staining in human NP samples assessed by Pearson‘s correlation analysis. (F and G) Western blot analyses of CEMIP, matrix metalloproteinase‐13 (MMP‐13), Collagen II and ACAN in degenerative and normal NP tissues. *n* = 3. (H and I) Cell immunofluorescence (IF) staining of CEMIP in degenerative and normal NP cells. Scale bar: 50 µm. *n* = 3. (J) Plasma levels of CEMIP in IDD patients and the controls, as detected by enzyme‐linked immunosorbent assay (ELISA). *n* = 6. (K) Correlation between CEMIP and the Pfirrmann scores of IDD patients assessed by Pearson‘s correlation analysis. ^*^ *p* < .05, ^**^ *p* < .01, ^***^ *p* < .001, ^****^ *p* < .0001.

CEMIP, a protein recognised for its role in HA depolymerisation within the ECM,^25^ was first identified in patients with non‐syndromic hearing loss^26^ and later reported in oncogenesis.^27^ Despite documented ageing‐related increases in CEMIP levels across skin, cartilage and bone tissues,^28^, ^29^, ^30^ its function in IDD has been less clear. Since integrative analysis of RNA‐seq and ATAC‐seq data suggested CEMIP's involvement in IDD, we then analysed the expression of CEMIP in NP tissue samples derived from stenosis patients and normal controls (Figure 4A). Histological examination of serial NP sections—including haematoxylin and eosin (HE), Safranin O‐fast green and Masson's trichrome staining—revealed increased CEMIP expression in degenerated tissues (Figure 4B), supported by IHC (Figure 4C,D), Western blot (Figure 4F,G) and IF assays (Figure 4H,I). Correlating with ECM disruption—a hallmark of IDD^31^—we noted decreased anabolic proteins ACAN and Collagen II, contrasted with elevated catabolic markers MMP‐9 and ADAMTS‐5 (Figure 4C,D). Interestingly, the CEMIP+ cells (%) of IHC staining exhibited significant correlations with that of ACAN+ cells (*p* = 0.0355) and Collagen II+ cells (*p* = .0406), suggesting a potential link between CEMIP levels and NP tissue degeneration (Figure 4E).

Since CEMIP could be detected in peripheral blood,^30^ the plasma levels of CEMIP were determined in IDD patients and normal control patients by enzyme‐linked immunosorbent assay (ELISA) to test for clinical relevance. We found that plasma CEMIP levels were higher in IDD patients compared to fracture controls (Figure 4J), highlighting CEMIP's potential as a biomarker. A significant positive correlation between plasma CEMIP levels and Pfirrmann grading of disc degeneration (*R* ^2^ = .4206, *p* = .0005) was observed (Figure 4K), further supporting its value in assessing IDD severity. These findings indicate CEMIP not only as a contributor to IDD pathophysiology but also as a promising candidate for diagnostic assessment.

### 3.5 CEMIP KO mice exhibit alleviated IDD

**FIGURE 5:**
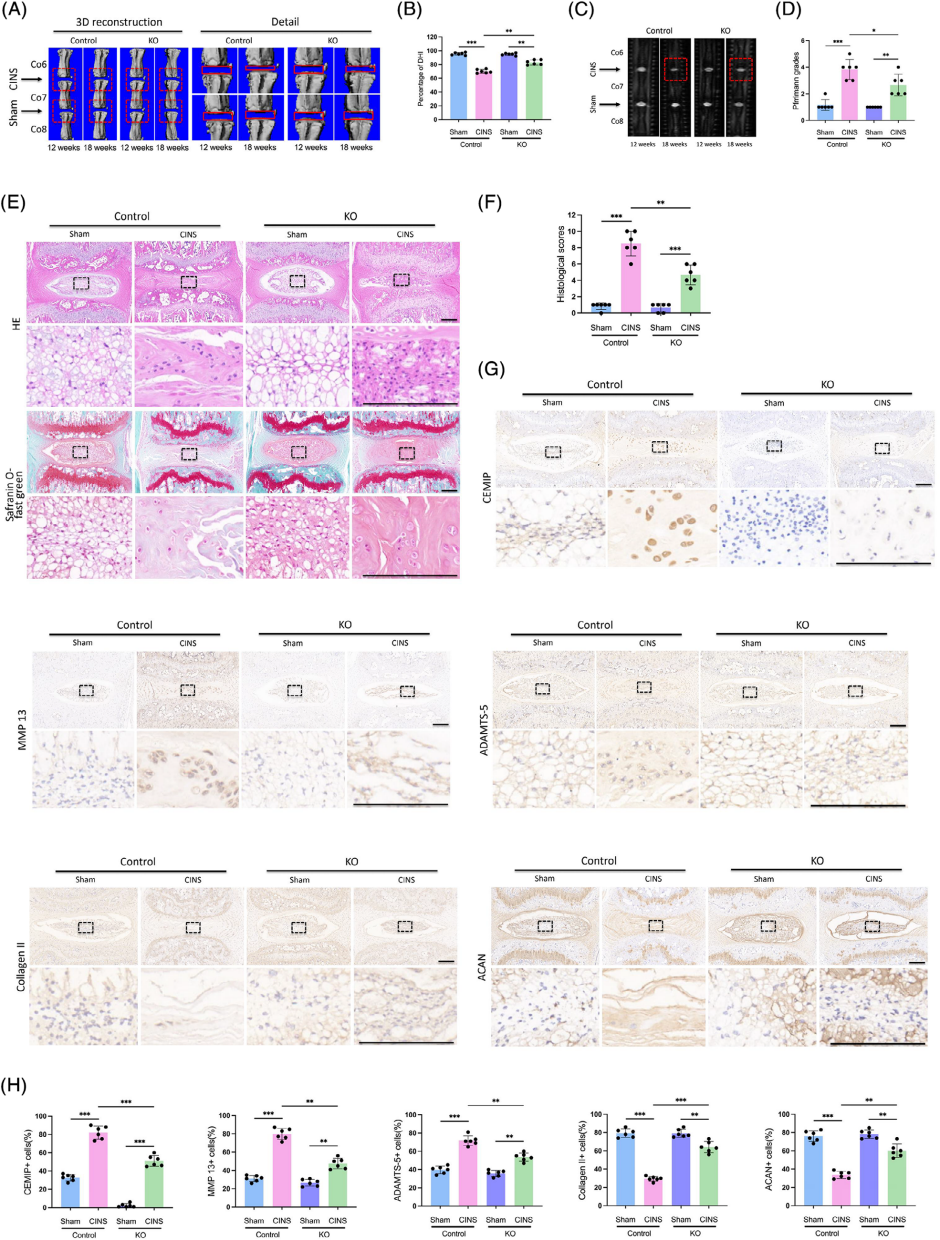
Cell migration‐inducing protein (CEMIP) knockout (KO) mice exhibit alleviated intervertebral disc degeneration (IDD). (A and B) Three‐dimensional micro‐computed tomography (µCT) reconstructions of coccygeal intervertebral discs (IVDs) in wild‐type (WT) and CEMIP KO mice with or without coccygeal intervertebral disc needle stab (CINS). Changes in the disc height index (%DHI) (Co6–7, 7–8) were evaluated by µCT analyses. *n* = 6. (C and D) Magnetic resonance imaging (MRI) and Pfirrmann grades of coccygeal IVDs in mice treated as in (A). *n* = 6. (E and F) Haematoxylin and eosin (HE) and Safranin O‐fast green staining and histological scores of coccygeal IVDs in WT and KO mice with or without CINS. Scale bar: 200 µm. *n* = 6. (G and H) Immunohistochemistry (IHC) staining of CEMIP, matrix metalloproteinase‐13 (MMP‐13), a disintegrin and metalloproteinase with thrombospondin motifs subtype 5 (ADAMTS‐5), Collagen II and Aggrecan (ACAN) in NP tissues in coccygeal IVDs of WT and KO mice with or without CINS. Scale bar: 200 µm. *n* = 6. ^*^ *p* < .05, ^**^ *p* < .01, ^***^ *p* < .001.

To elucidate the in vivo role of CEMIP in the IVD, we generated CEMIP KO mice employing CRISPR/Cas9‐mediated genome editing technology. We utilised a coccygeal intervertebral discs needle stab (CINS) model, in which the operated coccygeal IVDs were subjected to damage and aberrant mechanical stress due to the altered mechanics of the neutral zone.^32^ µCT scanning suggested a markedly reduced DHI in the CINS model of WT mice. This disc space collapse was mitigated in CEMIP KO mice, indicating an improved IVD degeneration (Figure 5A,B). T2‐weighted MRI supported these findings, displaying higher signal intensity in the IVDs of CEMIP KO mice, which suggests a synthetic ECM in the NP tissues of KO mice (Figure 5C,D).

Histological evaluations, including HE and Safranin O‐fast green staining, presented a decline in NP cell density within the WT CINS model, evidenced by disorganised annulus fibrosus lamellae and collapsed cartilage endplates (Figure 5E). In contrast, CEMIP KO mice exhibited markedly improved histological features in the CINS model (Figure 5E,F). IHC analysis provided further insight: ECM homeostasis was notably disrupted in WT mice post‐CINS, as characterised by reduced ACAN and Collagen II levels, alongside increased expression of matrix‐degrading enzymes MMP‐13 and ADAMTS‐5, with concurrent upregulation of CEMIP (Figure 5G,H). However, the IVDs of CEMIP KO mice revealed a significantly different status, with enhanced ACAN and Collagen II expression and reduced MMP‐13 and ADAMTS‐5 levels (Figure 5G,H). This suggests that the absence of CEMIP inhibits ECM degradation, conferring a protective effect against IVD degeneration.

Altogether, the evidence from µCT, MRI and histological assessments underscores CEMIP's critical role in maintaining ECM equilibrium in NP tissues. Our findings not only elucidate CEMIP's involvement in the pathogenesis of IDD but also reveal it as a promising target for therapeutic intervention.

### 3.6 CEMIP deficiency leads to the attenuation of IDD by inhibiting the degradation of HA

**FIGURE 6:**
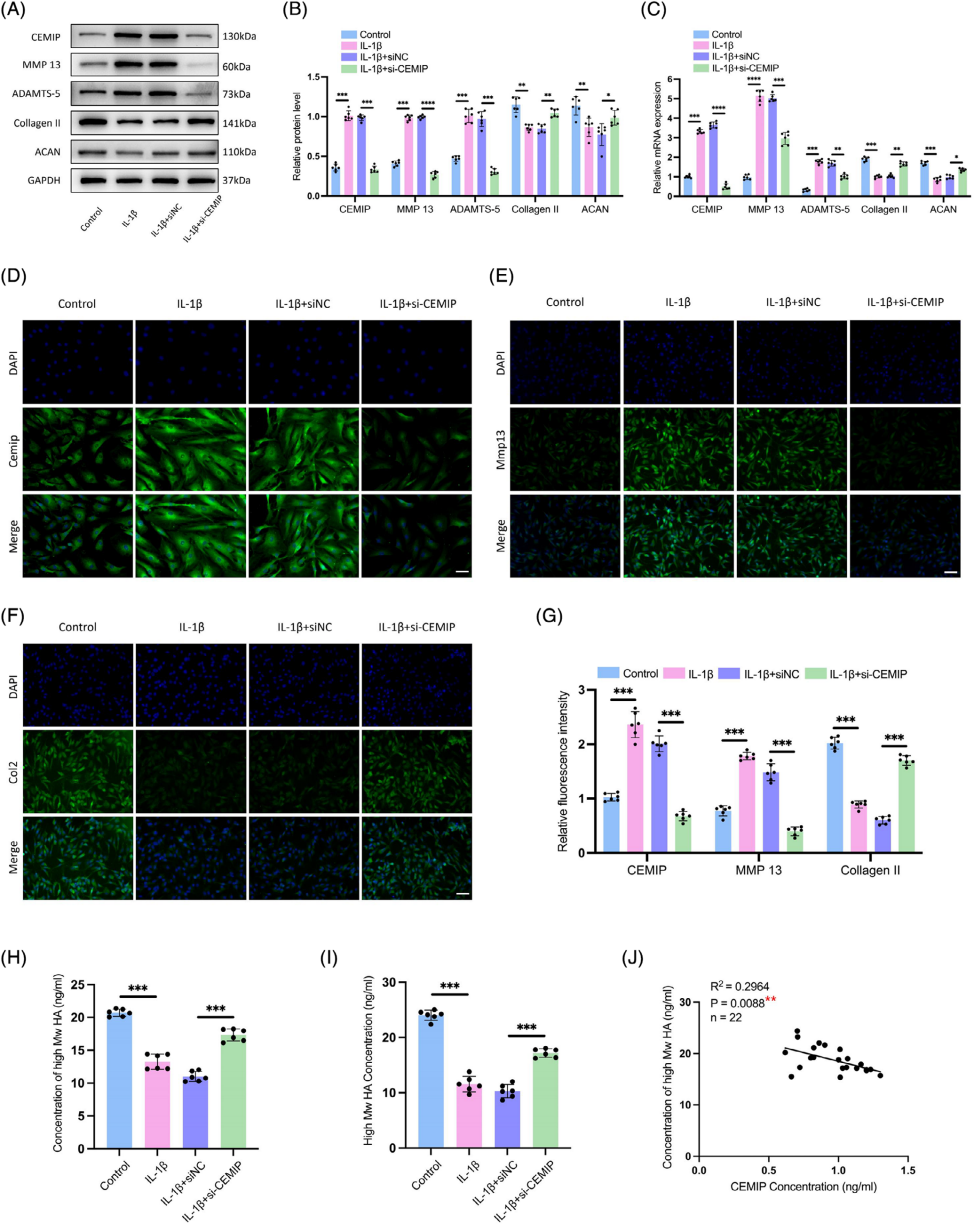
Cell migration‐inducing protein (CEMIP) deficiency leads to attenuation of intervertebral disc degeneration (IDD). (A and B) Western blot analyses of CEMIP, matrix metalloproteinase‐13 (MMP‐13), a disintegrin and metalloproteinase with thrombospondin motifs subtype 5 (ADAMTS‐5), Collagen II and Aggrecan (ACAN) in nucleus pulposus cells (NPCs), which were transfected with negative control siRNA (si‐NC) or CEMIP siRNA (si‐CEMIP), and then treated with or without interleukin‐1β (IL‐1β). *n* = 6. (C) qRT‐PCR analyses of CEMIP, MMP‐13, ADAMTS‐5, Collagen II and ACAN treated as in (A). *n* = 6. (D‒G) Cell immunofluorescence (IF) staining of CEMIP, MMP‐13 and Collagen II in NPCs treated as in (A). Scale bar: 50 µm. *n* = 6. (H and I) Concentration of high molecular weight hyaluronic acid (HMW‐HA) from NPCs culture medium treated as in (A), measured and quantified by enzyme‐linked immunosorbent assay (ELISA) (H) and high‐performance liquid chromatography (HPLC) (I). *n* = 6. (J) Correlation between the concentration of HMW‐HA and CEMIP assessed by Pearson‘s correlation analysis. ^*^ *p* < .05, ^**^ *p* < .01, ^***^ *p* < .001, ^****^ *p* < .0001.

To further explore CEMIP's role in NPC degeneration, siRNA‐mediated knockdown of CEMIP was performed in primary NPCs derived from IDD patients (Figure S3). Subsequently, IL‐1β was administered to establish an in vitro model of NPC degeneration to further explore the underlying mechanism of CEMIP.^33^ This model mimics the cellular phenotypic changes associated with IDD, such as decreased expression of ACAN and Collagen II, coupled with increased levels of MMP‐13 and ADAMTS‐5 (Figure 6A‒C). Upon CEMIP depletion, we observed a significant reversal in this trend: Collagen II and ACAN levels increased, while MMP‐13 and ADAMTS‐5 expressions were notably decreased (Figure 6A‒C). IF assays confirmed these findings, displaying increased Collagen II and decreased MMP‐13, indicating CEMIP's important anti‐catabolic effect on ECM dynamics in IL‐1β‐treated NPCs (Figure 6E‒G). These results indicated a shift away from catabolic ECM degradation towards anabolic activity upon CEMIP silencing.

As a known regulator of ECM homeostasis and a hyaluronidase, CEMIP's involvement in HA degradation is well‐documented. Building on previous research showing CEMIP's role in degrading HMW‐HA into low molecular weight hyaluronan (LMW‐HA) fragments,^34^ we investigated this activity in NPCs. HA's biological function is linked to its molecular size; with HMW‐HA typically presenting anti‐inflammatory and anti‐angiogenic effects, while LMW‐HA is associated with pro‐inflammatory, pro‐angiogenic and promotion of cell migration.^35^ Through HPLC, we quantified HA molecular weights in NPC culture mediums^36^ (Figure S5). Following IL‐1β induction, a decline in HMW‐HA levels coincided with increased CEMIP activity; yet, this trend was reversed with CEMIP silencing, restoring HMW‐HA to baseline concentrations measured by ELISA and HPLC (Figure 6H,I). Moreover, a direct correlation was established between CEMIP and HMW‐HA levels (*R* ^2^ = .2964, *p* = .0088) in cell supernatants, reinforcing the link between CEMIP expression and HA molecular size (Figure 6J). Altogether, these findings suggested that the CEMIP mediated HA degradation in NPCs, releasing HA fragments into the ECM. Thus, silencing of CEMIP may lead to attenuation of IDD by inhibiting the degradation of HA and subsequently rebalancing the ECM metabolism.

### 3.7 Highly expressed CEMIP induces a fibrosis‐like change in NP tissues

**FIGURE 7:**
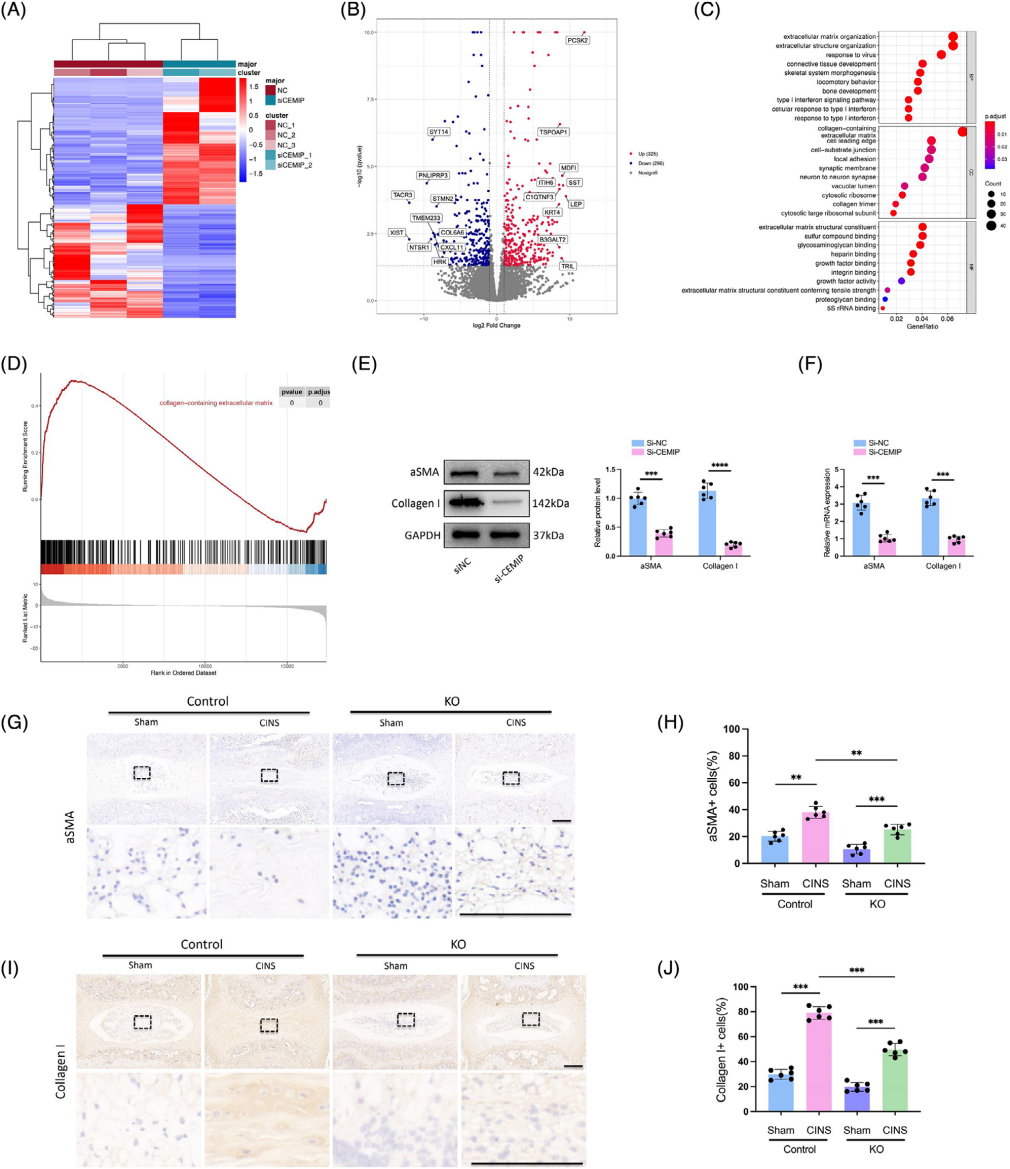
Highly expressed cell migration‐inducing protein (CEMIP) induces a fibrosis‐like change in NP tissues. (A) Heatmap of the most regulated genes identified by RNA sequencing (RNA‐seq) in CEMIP‐deficient human nucleus pulposus cells (NPCs) (degenerated NPCs transfected with specific si‐CEMIP), compared to non‐target siRNA transfected control NPCs (si‐NC). (B) Volcano plot comparing differentially expressed genes (DEGs) in si‐CEMIP NPCs to control NPCs. (C) Gene Ontology (GO) enrichment analysis for differential genes. (D) Gene set enrichment analysis (GSEA) analysis showed a significantly enriched pathway related to the expression of collagen after CEMIP knockdown. (E and F) Expression levels of alpha‐amooth muscle actin (α‐SMA) and Collagen I were traced by Western blot (E) and qRT‐PCR (F) in si‐CEMIP NPCs and control NPCs. (G‒J) Immunohistochemistry (IHC) staining of α‐SMA (G and H) and Collagen I (I and J) in NP tissues in coccygeal intervertebral discs (IVDs) of wild‐type (WT) and CEMIP knockout (KO) mice with or without CINS. Scale bar: 200 µm. *n* = 6. ^**^ *p* < .01, ^***^ *p* < .001, ^****^ *p* < .0001.

**FIGURE 8:**
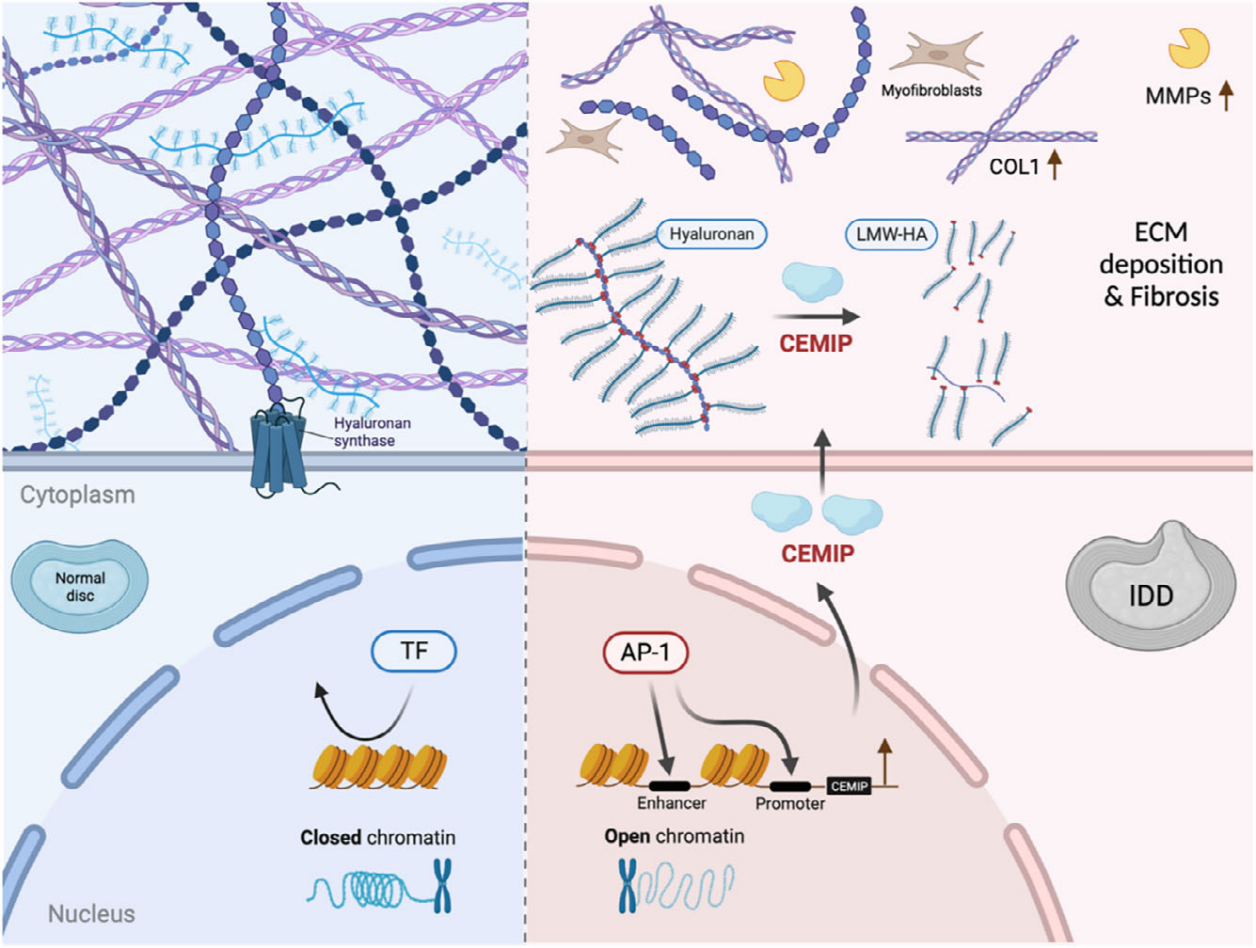
Proposed schematic depicting the mechanism of the regulatory chromatin landscape governing nucleus pulposus cells (NPCs) degeneration, highlighting cell migration‐inducing protein (CEMIP) accelerating intervertebral disc degeneration (IDD) and identifying the activator protein‐1 (AP‐1) family as a critical modulator. In degenerated NPCs, AP‐1 activation increased the accessibility of chromatin to transcriptionally upregulate CEMIP, while CEMIP disrupted extracellular matrix (ECM) (such as Aggrecan [ACAN] and Collagen II) homeostasis through its regulation of high molecular weight hyaluronic acid (HMW‐HA) degradation, and its contribution to fibrotic changes within NP tissues. The AP‐1/CEMIP axis emerges as a novel target for the prevention and treatment of IDD.

To investigate how CEMIP could regulate the features of NPCs, the mRNA of NPCs was profiled after CEMIP depletion using siRNA and compared to the control cells. Analysis revealed alterations in gene expression: 296 genes were downregulated, while 325 were upregulated in CEMIP‐depleted NPCs compared to controls (*p*‐value adjusted < .05 and |fold change| > 2, Figure 7A,B). GO analysis identified significant alterations in signalling pathways related to ECM upon CEMIP depletion, with ‘extracellular matrix organisation’ and ‘extracellular structure organisation’ being the markedly enriched processes (Figure 7C and Table S3). GSEA further highlighted the dominance of ECM‐associated pathways, particularly those involving collagen: ‘collagen‐containing extracellular matrix’, ‘collagen fibril organisation’ and ‘extracellular matrix structural constituent conferring tensile strength’ (Figures 7D and S2 and Table S4), substantiating CEMIP's influence on the ECM of NPCs. (Figure 8)

Since the ‘collagen‐containing extracellular matrix’ and ‘collagen fibril organisation’ pathways were enriched by the GSEA analysis and we found increased expression of Collagen II after knocking down CEMIP, we reasoned that the expression of Collagen I would change after CEMIP depletion. qRT‐PCR and Western blot confirmed that the expression of Collagen I was decreased in degenerated NPCs treated with si‐CEMIP compared to control NPCs (Figure 7E,F). This finding is significant as Collagen I fibrillar accumulation is a hallmark of fibrotic ECM. Additionally, the downregulation of pro‐fibrotic markers (such as COL6A6, XIST, CXCL11, FGF12, DKK1) in our RNA‐seq post‐CEMIP depletion supports a potential role for CEMIP in NP tissue fibrosis (Figure 7B and Table S5). This is further evidenced by the reduced mRNA and protein levels of α‐SMA, a fibrosis marker,^37^ in CEMIP‐depleted NPCs (Figure 7E,F).

Histological analysis of the IVDs in the CINS mice model confirmed the onset of fibrosis, marked by elevated Collagen I and α‐SMA expression at 6 weeks post‐CINS in WT mice, suggesting an injury‐induced shift of NP cells towards a fibroblast‐/myofibroblast‐like phenotype, in alignment with prior studies.^38^ In contrast, CEMIP KO mice exhibited reduced Collagen I and α‐SMA levels (Figure 7G‒J). Interestingly, CEMIP deficiency also reduced the expression of fibrotic markers in no‐CINS mice (Figure 7G‒J). The alleviated fibrosis in the CEMIP deficiency model suggests that matrix remodelling may be involved in functional compensation, or otherwise attempt to elicit a reparative process involving a granulation tissue state, which ultimately contributes to a fibrocartilaginous NP due to insufficient proper tissue regeneration.

## 4 DISCUSSION

IDD, a chronic condition linked to ageing, is characterised by NPCs degeneration and ECM remodelling. Here, through the integration of the accessible chromatin landscape and the transcriptional profile, we for the first time, identified CEMIP as an important molecular in NPC degeneration, regulated via an AP‐1‐dependent pathway. CEMIP, significantly upregulated in IDD‐derived NPCs, drives hyaluronic acid degradation, facilitating a catabolic state that culminates in the dysregulation of critical ECM components and advances NPC degeneration.

Our findings reveal an elevation of CEMIP not only in degenerated NPCs but also in blood plasma from IDD patients, suggesting a systemic manifestation of local tissue changes. This upregulation of CEMIP reflects pathological features observed in fibrotic diseases, rheumatoid arthritis and osteoarthritis, which share common inflammatory pathways. A recent study has demonstrated that the deficiency of CEMIP enhances the differentiation of skeletal stem cells into osteoblasts and promotes bone regeneration.^30^ The current study presents the vital role of CEMIP in IDD, as evidenced by our CEMIP KO mouse model, providing a mechanistic link to the ECM remodelling, particularly the altered expression of Collagen II and ACAN. CEMIP, a hyaluronidase‐like protein, plays an important role in the depolymerisation of HA, fragmenting HMW‐HA into pieces smaller than 10 kDa. CEMIP‐expressing cells have previously been shown to degrade exogenously added HA^34^, ^39^ and our study expands on the understanding of CEMIP's function by demonstrating its ability to hydrolyse endogenous HA. HMW‐HA is traditionally viewed as a cellular protector, forming a structural scaffold and interacting with cell surface receptors to maintain tissue homeostasis. Interestingly, LMW‐HA fragments, often resulting from CEMIP activity, have been shown to stimulate either defensive or pro‐inflammatory cellular responses in various cell types.^40^, ^41^, ^42^, ^43^ Interestingly, NPCs exposed to IL‐1β exhibited a reduced concentration of HMW‐HA, a process reversible by CEMIP knockdown. This suggests that CEMIP's enzymatic activity is a critical factor in the catabolic processing of HA within NPCs. The pathological implications of elevated CEMIP levels, similar to those found in osteoarthritis and rheumatoid arthritis, are notable for their potential to accumulate LMW‐HA, thus promoting a microenvironment beneficial to angiogenesis and inflammation. Such a scenario is validated by previous research identifying heightened CEMIP expression in the chondrocytes and synovial fibroblasts of osteoarthritis patients, implicating it as a driver of HA degradation and associated pathophysiological changes.^44^, ^45^

We have observed that CEMIP's upregulation induces a fibrotic transformation in NPCs, a shift supported by increased Collagen I expression and decreased Collagen II expression. This suggests that CEMIP could be an inducer of the NPCs dedifferentiation process leading to a fibroblastic‐like phenotype, which is critical in the pathogenesis of IDD. The fibrogenic influence of CEMIP, previously identified in osteoarthritis chondrocytes^45^ and synovial membranes,^44^ as well as its induction of fibrosis in Crohn's disease^46^ and idiopathic pulmonary fibrosis,^47^ emphasises its central role in diseases characterised by abnormal ECM remodelling and fibrosis. Myofibroblasts, expressing α‐SMA as a marker, are regarded as primary effector cell types in tissue fibrosis. Previous studies suggested that resident notochord‐derived NP cells can be an origin of myofibroblasts in the induced IDD model.^38^, ^48^ Our findings, which demonstrate concurrent expression of CEMIP and α‐SMA, suggest that NPCs might intrinsically switch towards myofibroblasts. This is supported by Deroyer et al.’s identification of ‘chondro‐myo‐fibroblasts’ (dedifferentiated chondrocytes sharing an expression pattern with activated myofibroblasts) in osteoarthritis, indicating a shared fibrotic expression profile inclusive of CEMIP expression.^45^ Furthermore, Tu et al. described six subpopulations of NPCs using single‐cell RNA‐seq, with one of the subpopulations being fibroNPCs expressing mRNAs that encode related with fibrosis, such as COL1A1, COL3A1 and COL6A1.^49^ Similarly, our RNA‐seq data also confirmed the altered expression of COL6A6 after knocking down CEMIP (Figure 7B). Tu's study aligns with our observations and reinforces the concept that CEMIP is a significant factor in the NPC fibrotic phenotype. Beyond fibrosis, CEMIP's role in the epithelial‐to‐mesenchymal transition (EMT) process across various tumor cell types^26^, ^50^, ^51^ connects it with a broader spectrum of pathological processes. Since EMT shares key molecular pathways with fibrosis, such as transforming growth factor beta 1 (TGF‐β1), tumor necrosis factor alpha (TNF‐α), nuclear factor kappa B (NF‐κB) and Snail signalling,^52^ understanding CEMIP's involvement in fibrosis is vital. This insight suggests a potential therapeutic angle, advocating for the exploration of anti‐EMT and anti‐fibrosis drugs as novel treatments for conditions such as IDD. Altogether, our research indicates CEMIP not only as a contributor to ECM remodelling but also as a therapeutic target. By unraveling the relationship between CEMIP and fibrosis within disc degeneration, we open the possibility of developing targeted treatments that address the underlying mechanisms of IDD progression.

Notably, our study has identified the AP‐1 family as a dominant regulator of CEMIP expression, driving the chromatin accessibility changes crucial for IDD progression. Bioinformatics analysis has uncovered a substantial enrichment of AP‐1 motifs, present in over half of the chromatin regions that gain accessibility in IDD‐derived NPCs. This finding aligns with a previous study demonstrating that accessible chromatin rearrangement determines the gene expression profile in senescent cells.^53^ Works by Zhang et al. and others^54^, ^55^, ^56^ have proved the AP‐1 family as responsible for driving chromatin accessibility reconstruction in IARs during cell senescence and regeneration post‐injury, suggesting a complex response mechanism where AP‐1 activation precipitates genome‐wide chromatin alterations in response to tissue damage. Extending these findings, we demonstrate the involvement of c‐Fos, a component of the AP‐1 family, in regulating IAR accessibility, thus changing the CEMIP expression during the IDD process. While AP‐1 TFs have been established as central players in modulating chromatin structure, our data suggest that the specific AP‐1 subunits active in this process may differ across cell types and pathological conditions.^53^, ^57^ This underscores the necessity of a deeper exploration of AP‐1 family functions, to identify precise intervention targets to counteract degenerative processes, particularly in IDD.

Previous reports have established a connection between the AP‐1 activity and IDD. Li et al. suggested that IL‐17A enhances the expression of COX2 in NPCs through the activation of p38/c‐Fos and JNK/c‐Jun signalling pathways.^24^ Makino et al. demonstrated that the selective c‐Fos/AP‐1 inhibitor T‐5224 can suppress the catabolic factors induced by c‐Fos/AP‐1 in the IDD model, alleviating the disc degeneration.^58^ However, the downstream effectors of AP‐1 in IDD remained unexplored until our study, which illustrates that AP‐1 accelerates NPC degeneration through the upregulation of CEMIP. Our results align with breast cancer study^59^ and colorectal cancer study^60^ that have found CEMIP expression to be regulated by NF‐κB and AP‐1‐binding sites within its promoter region, indicating that the interplay between genetic and epigenetic mechanisms is crucial for its gene expression. Specifically, our study identifies c‐Fos as a dominant driver of CEMIP expression in degenerated NPCs, suggesting a new avenue for therapeutic intervention targeting the AP‐1/CEMIP axis to mitigate IDD progression.

In the IDD mice model, CEMIP‐deficient mice exhibit higher expression of anabolic factors and signs of mitigated disc degeneration. This evidence of CEMIP's deficiency leading to improved disc health strengthens its potential as a therapeutic target in treating IDD. Encouragingly, several studies have already examined CEMIP as a therapeutic target for different diseases. For instance, emerging preclinical studies have initiated the exploration of strategies aimed at inhibiting the HA‐degrading function of CEMIP, such as in models of skin ageing,^61^, ^62^ while early‐phase investigations in osteoarthritis synovial membranes suggest that reducing CEMIP expression may mitigate inflammation.^44^ Although these findings highlight CEMIP's potential as a therapeutic target, they remain preliminary and require further validation in clinically relevant models of IDD. Importantly, translating these approaches to disc degeneration will require addressing challenges specific to the IVD microenvironment, including limited vascularisation and the complex crosstalk between inflammatory and fibrotic pathways. However, this accumulating research suggests that modulating CEMIP activity, either through small‐molecule inhibitors or CEMIP‐specific neutralising antibodies, could be a fruitful direction for therapeutic development. Such strategies, by targeting the molecular foundations of IDD, may offer novel treatments to alleviate or reverse the disc degeneration process.

While our findings nominate the AP‐1/CEMIP axis as a promising therapeutic target for IDD, we emphasise the need to critically address associated challenges. First, the pleiotropic roles of AP‐1 TFs across physiological processes raise concerns about unintended systemic effects upon modulation. Second, the intricate crosstalk between multiple cell types and signalling mediators within fibrotic pathways complicates targeted intervention. Third, therapeutic efficacy may be constrained by compensatory mechanisms: notably, functional redundancy among HA‐degrading enzymes and cellular adaptations to CEMIP inhibition could bypass therapeutic blockade. Although CEMIP represents a mechanistically grounded target for IDD, its translational development demands systematic investigation of these biological and pharmacological complexities. We are committed to furthering our research in this area and will continue to explore the feasibility and safety of targeting CEMIP as a therapeutic approach for IDD.

In conclusion, our research reveals a complex interplay between chromatin architecture and gene expression regulation during NPC degeneration. We have discovered that AP‐1 TFs, particularly c‐Fos, are involved in disc degeneration. They achieve this by modulating chromatin accessibility within IARs, thereby enhancing the expression of CEMIP, a key gene implicated in the degenerative cascade. The elucidation of AP‐1 as an important regulator of CEMIP expression and chromatin remodelling establishes a promising avenue for therapeutic intervention in IDD. These insights offer a fresh perspective on IDD pathophysiology and present novel molecular targets that may revolutionise therapeutic strategies for this debilitating disease.

## AUTHOR CONTRIBUTIONS

*Conceptualisation*: Shibin Shu and Hongda Bao. *Methodology*: Xin Zhang, Shibin Shu and Hongda Bao. *Investigation*: Shibin Shu, Xin Zhang, Zhenhua Feng, Fengrui Li, Yating Wu and Hongda Bao. *Writing—original draft*: Shibin Shu and Hongda Bao. *Writing—review and editing*: Shibin Shu and Hongda Bao. *Funding acquisition*: Yong Qiu and Hongda Bao. *Resources*: Zhen Liu, Bo Shi and Kaiyang Wang. *Supervision*: Yong Qiu, Zezhang Zhu and Hongda Bao. All authors contributed to interpret the results.

## CONFLICT OF INTEREST STATEMENT

The authors declare they have no conflicts of interest.

## ETHICS STATEMENT

This study was conducted in accordance with the ethical guidelines approved by the Ethics Committee of Nanjing Drum Tower Hospital, Affiliated Hospital of Nanjing University Medical School (approval no. 2023‐506‐01). Informed consent was obtained from all participants.

## Supporting information



Supporting information

Supporting information

Supporting information

Supporting information

Supporting information

Supporting information

## Data Availability

All data relevant to this study are included in the article or uploaded as supplemental information.
