# Supplementary material for: Upregulated CEMIP promotes intervertebral disc degeneration via AP‐1‐mediated change in chromatin accessibility
Source: Clin Transl Med. 2025 May 21;15(5):e70322. doi: 10.1002/ctm2.70322 (PMC12095184; doi:10.1002/ctm2.70322)
Supplement: Supplementary file 1 — Supporting information [file CTM2-15-e70322-s006.docx]

**Supplemental Information**

**Up-regulated CEMIP Promotes Intervertebral Disc Degeneration Via AP-1-mediated Change in Chromatin Accessibility**

**Short running title: CEMIP via AP-1-mediated Chromatin Change in IDD**

Shibin Shu, phD^1^, Xin Zhang, phD^1^, Zhenhua Feng, phD^1^, Zhen Liu, phD^1^, Kaiyang Wang, phD^1^, Fengrui Li, MS^1^, Yating Wu, MS^1^, Bo Shi, phD^1^, Yong Qiu, MD^1^*, Zezhang Zhu, MD^1^*, Hongda Bao, phD^1^*

^1^Division of Spine Surgery, Department of Orthopedic Surgery, Nanjing Drum Tower Hospital, Affiliated Hospital of Medical School, Nanjing University, Nanjing, China.

* Corresponding authors.

Hongda Bao, Email: baohongda123@gmail.com

Zezhang Zhu, Email: [zhuzezhang@126.c](mailto:zhuzezhang@126.com)om

Yong Qiu, Email: [scoliosis2002@sina.com](mailto:scoliosis2002@sina.com)

Address: Division of Spine Surgery, Department of Orthopedic Surgery, Nanjing Drum Tower Hospital, Affiliated Hospital of Medical School, Nanjing University, Zhongshan Road 321, Nanjing 210008, China;

Tel./ Mobile Number: +86-021-83106666

Fax: +86-021-83106666

Fig. S1.


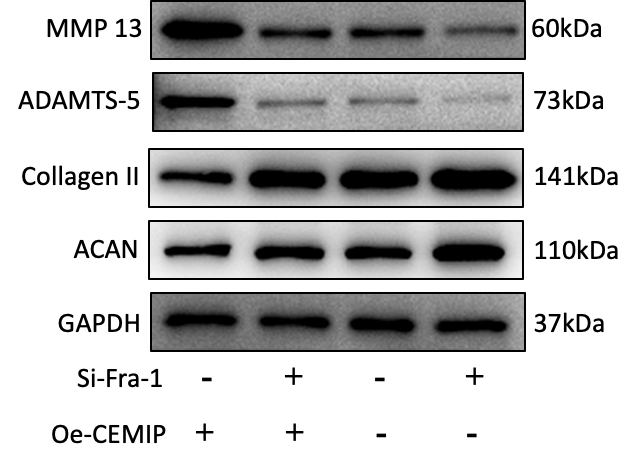


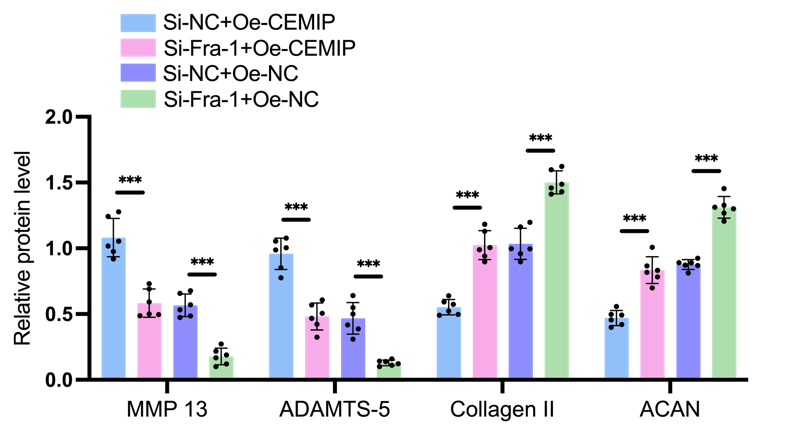


Fig. S1. Western blotting analyses of Mmp13, Adamts5, Col2a1 and Acan in NP cells, which were transfected with si-NC or si-Fra-1, and then treated with or without Oe-CEMIP. n = 6. ***P < 0.001

Fig. S2.


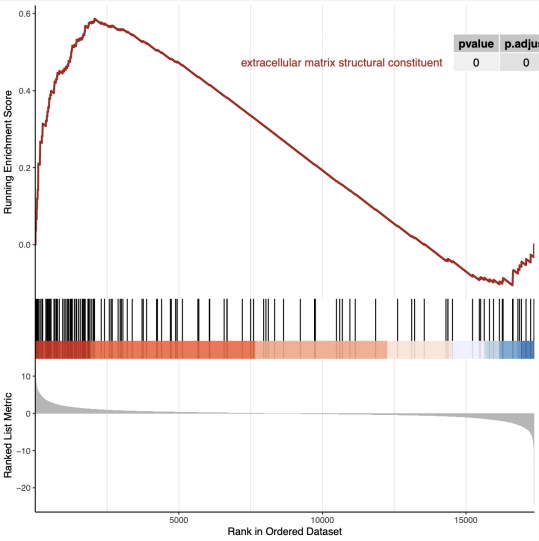

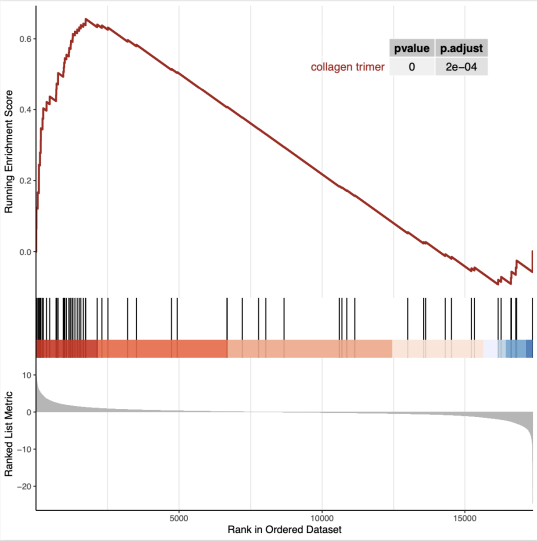

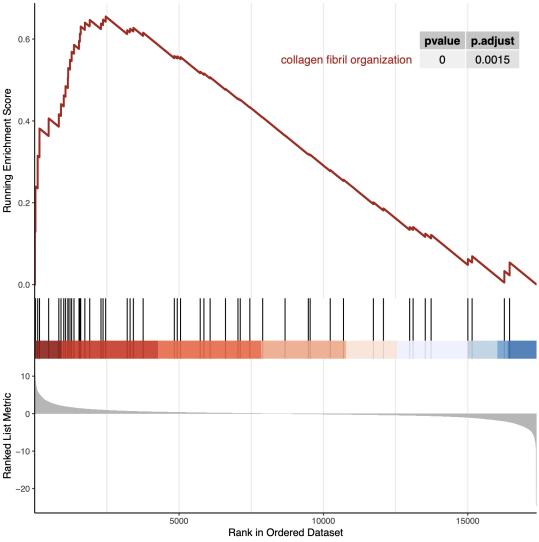

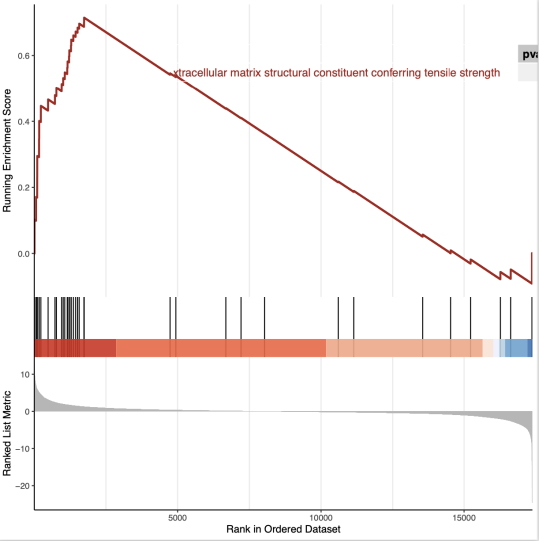


**A**

**B**

**C**

**D**

Fig. S2. Gene Set Enrichment Analysis (GSEA) analysis showed significantly enriched pathway related to expression of collagen after CEMIP knockdown.

Fig. S3.


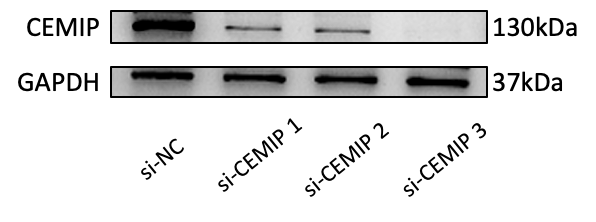

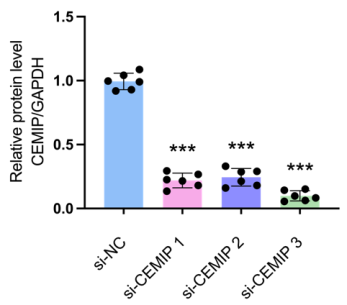


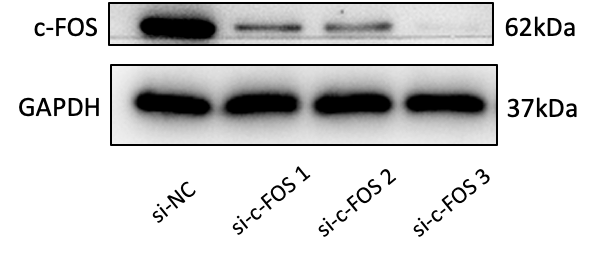

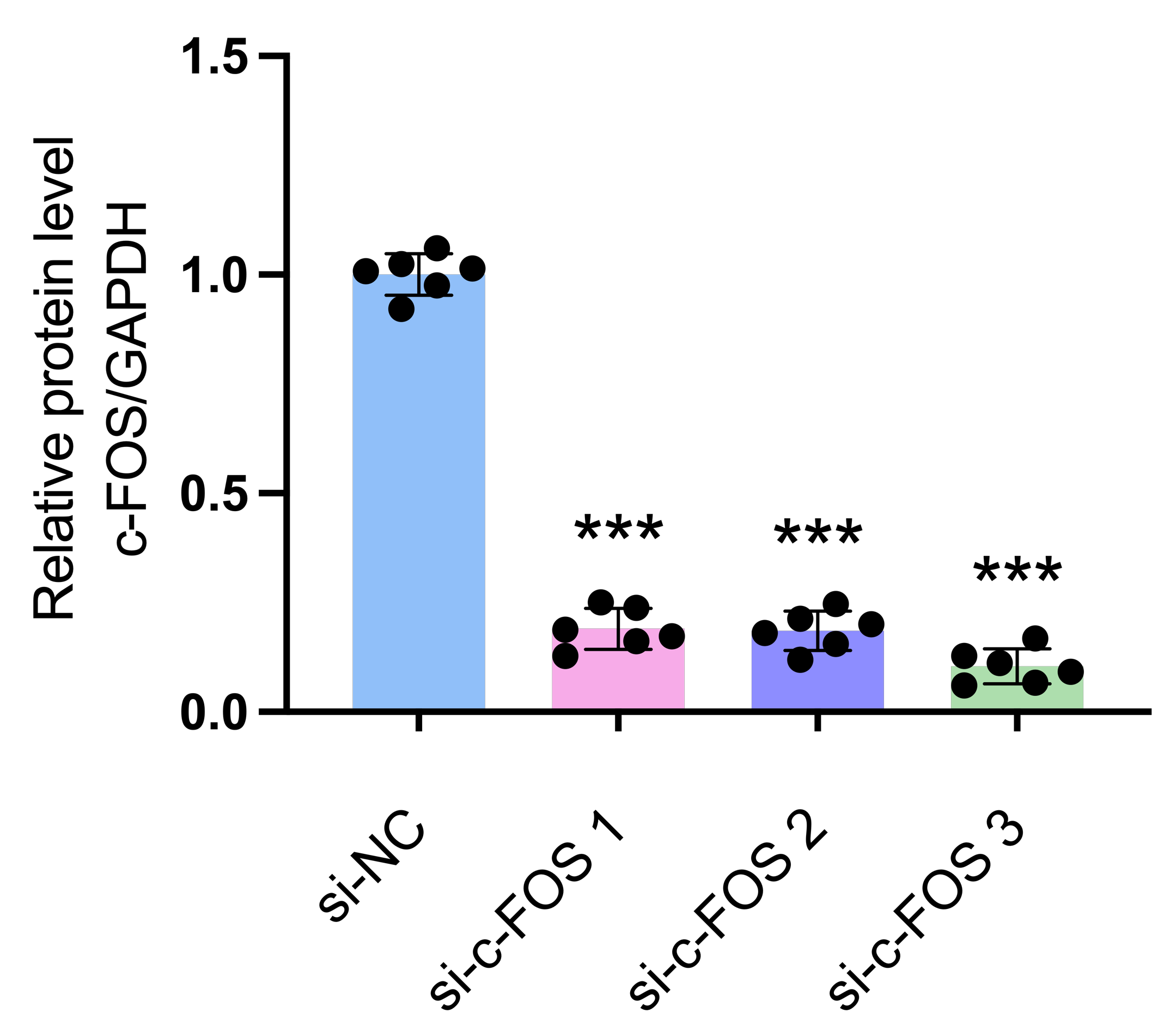


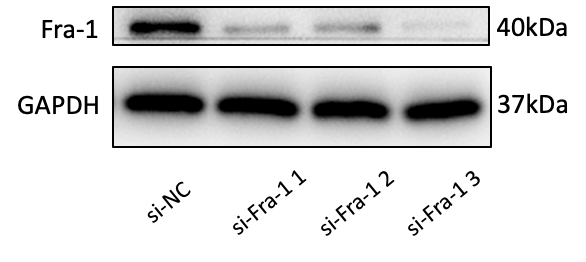

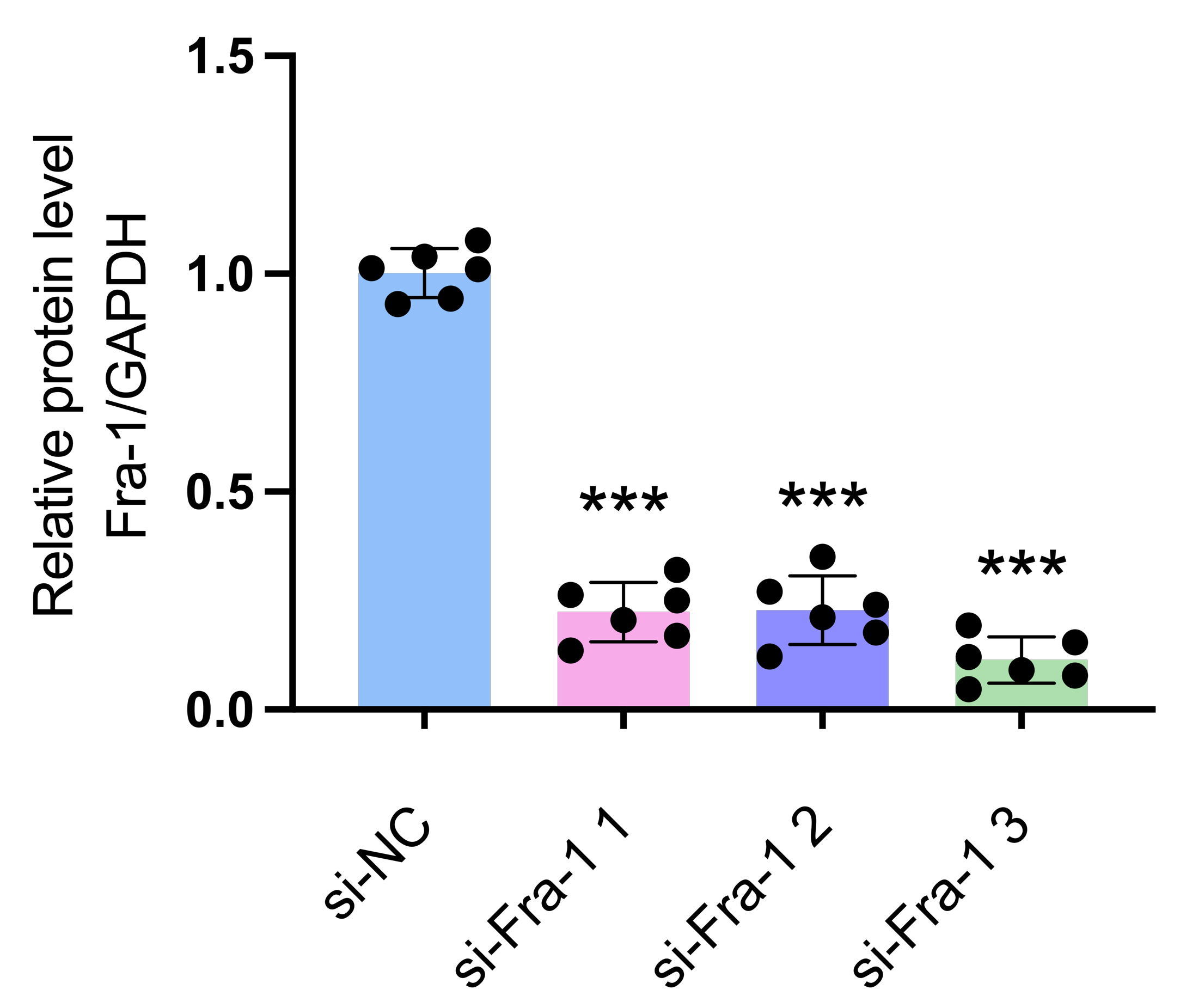


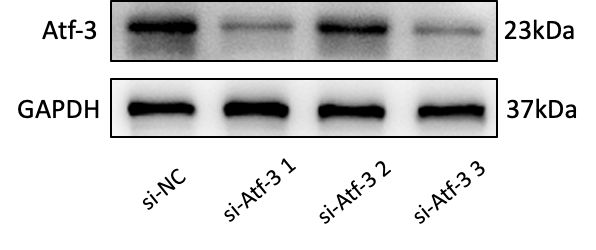

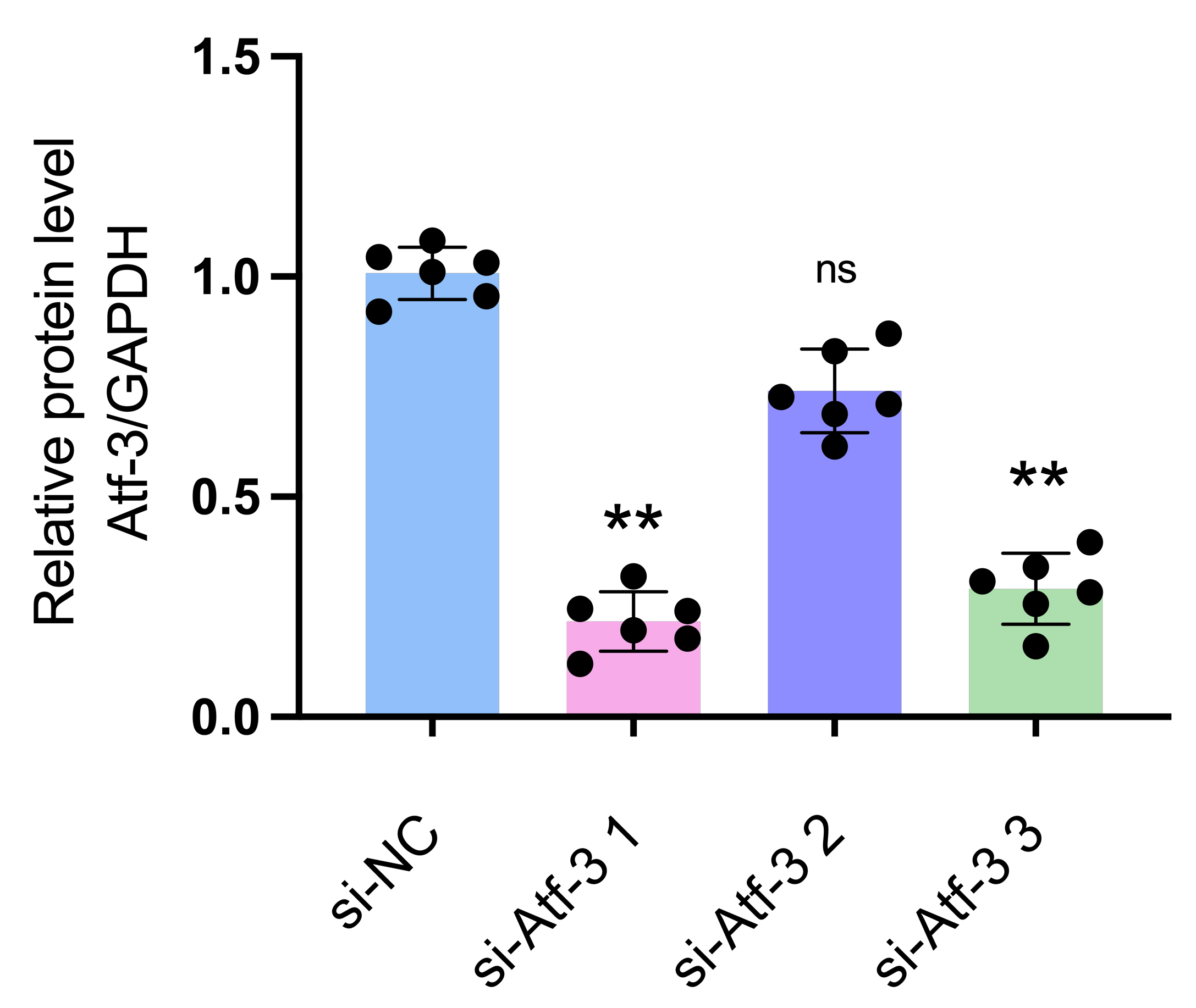


Fig. S3. The most effective target sequence for siRNA. Western blotting analyses of CEMIP, c-FOS, Fra-1 and Atf-3 in nucleus pulposus (NP) cells transfected with negative control (NC) siRNA and three independent siRNAs. si-CEMIP #3,si-c-FOS #3, si-Fra-1 #3, and si-Atf-3 #1 were the most effective target sequence for siRNA. n = 6. NS, no statistical significance, **P < 0.01, ***P < 0.001

Fig. S4.


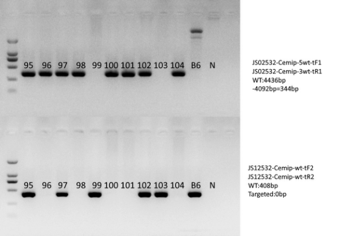


Fig. S4. Genotyping. Genotyping using tail DNA.

Fig. S5.


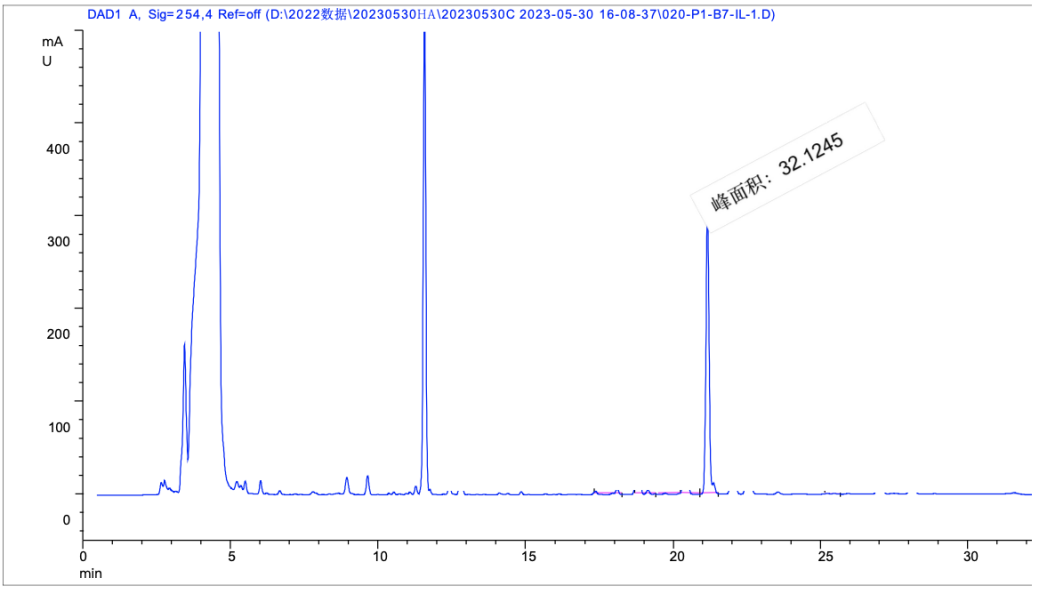


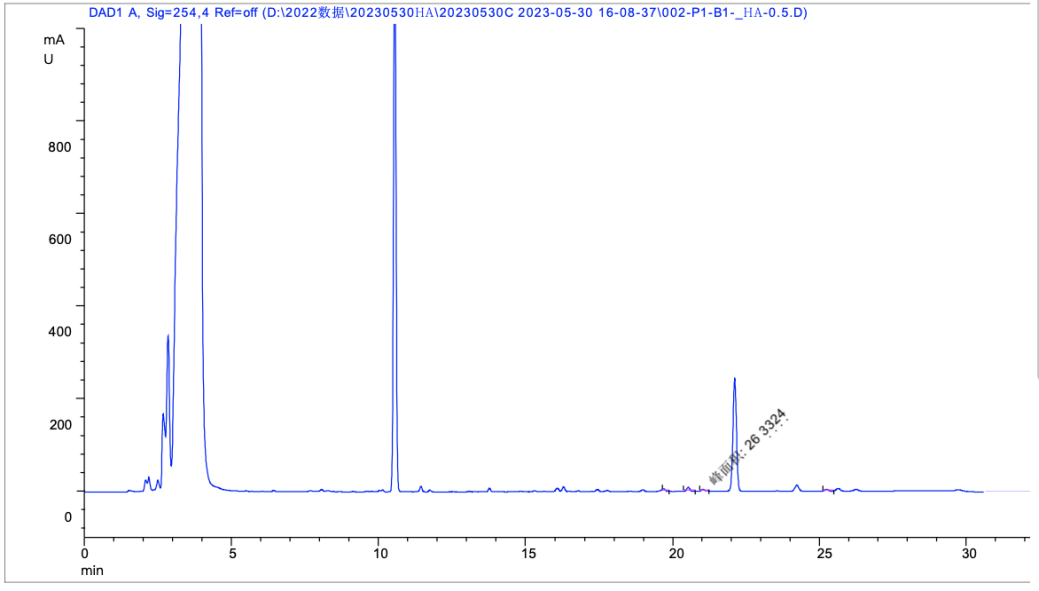


Fig. S5. Working chromatogram of HPLC

Fig. S6.


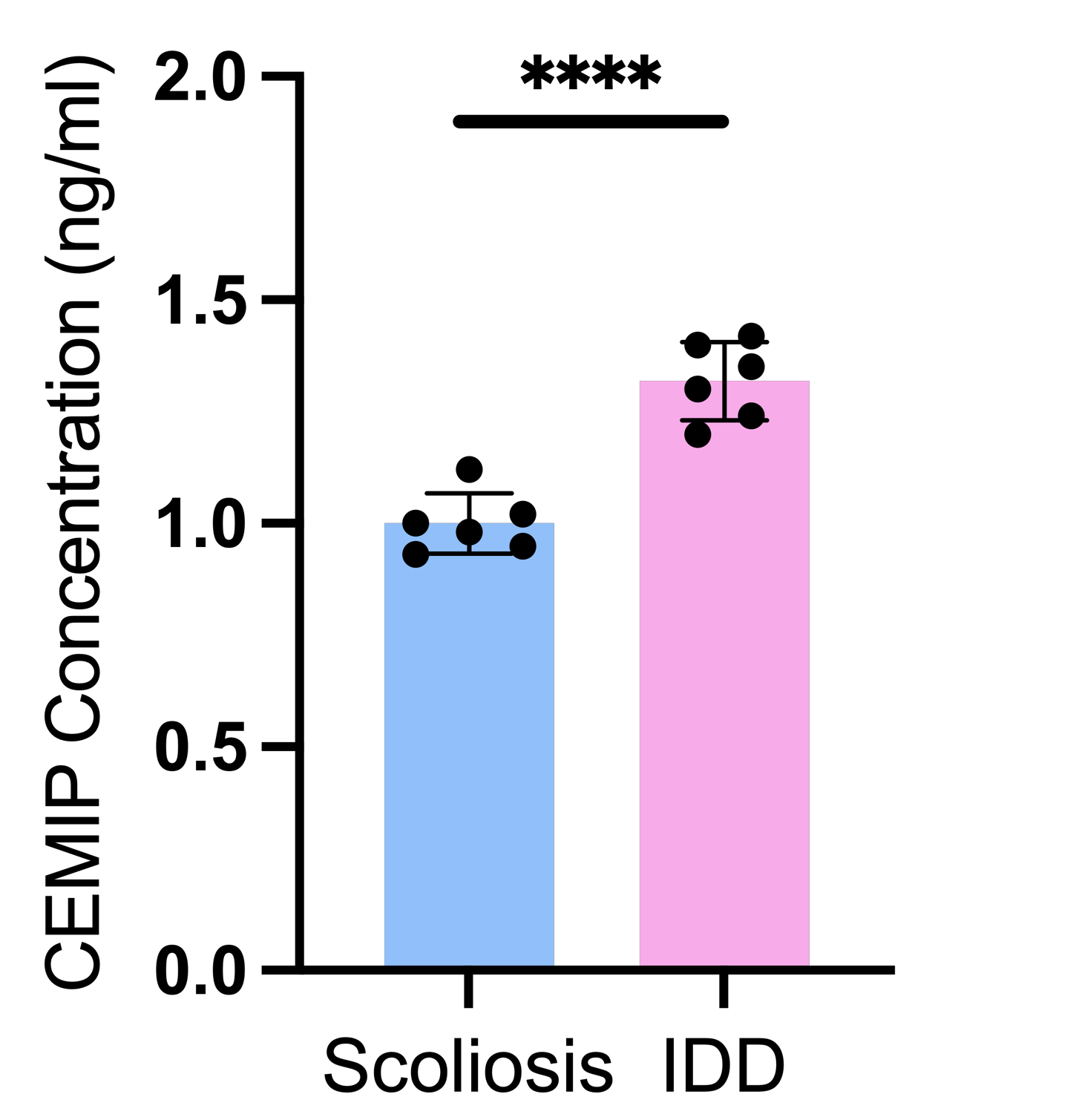


**Fig. S6. CEMIP is highly expressed in the blood circulation of IDD patients.**  The plasma levels of CEMIP in IDD patients and scoliosis patients, as detected by Enzyme-linked immunosorbent assay (ELISA). n = 6. ****P < 0.0001

Fig. S7.

**
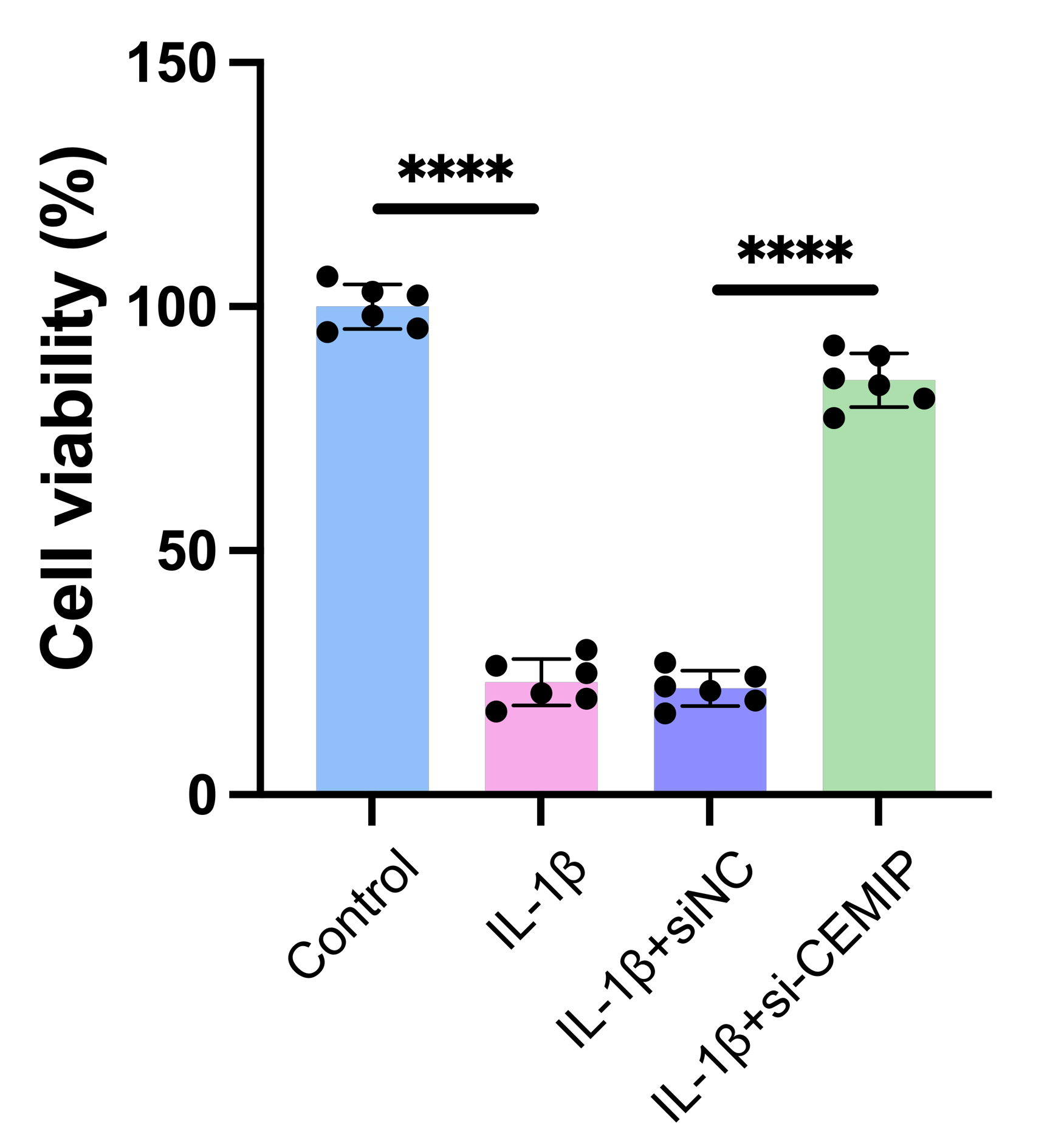
**

**Fig. S7.** The results of CCK-8 assay in NP cells, which were transfected with si-NC or si-CEMIP, and then treated with or without IL-1β. n = 6. ****P < 0.0001

Fig. S8.


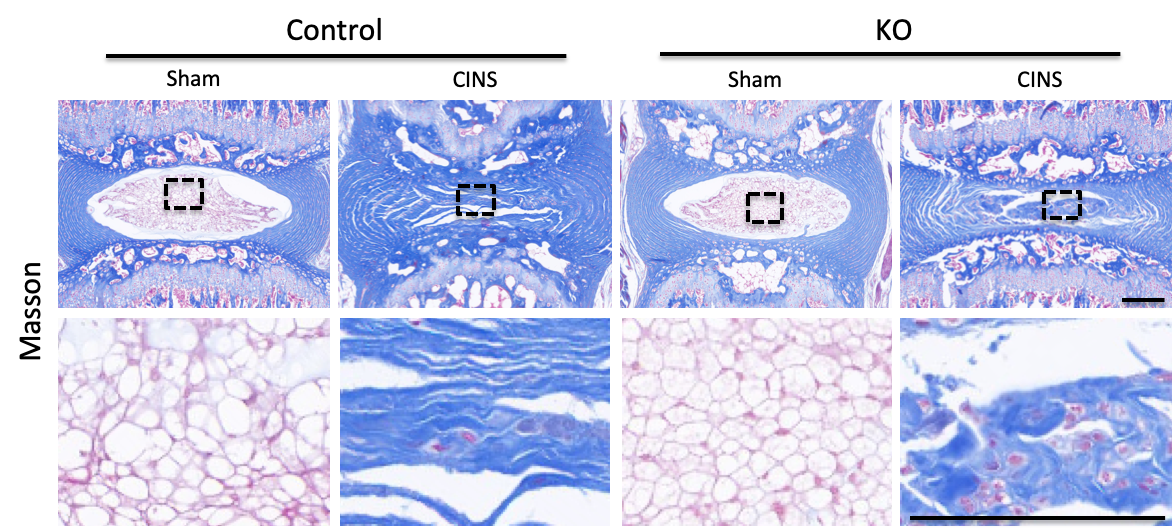


**Fig. S8. CEMIP KO mice exhibit alleviated fibrosis.** Masson's trichrome staining in NP tissues of WT and CEMIP KO mice with or without CINS. Scale bar, 200μm.

Fig. S9.


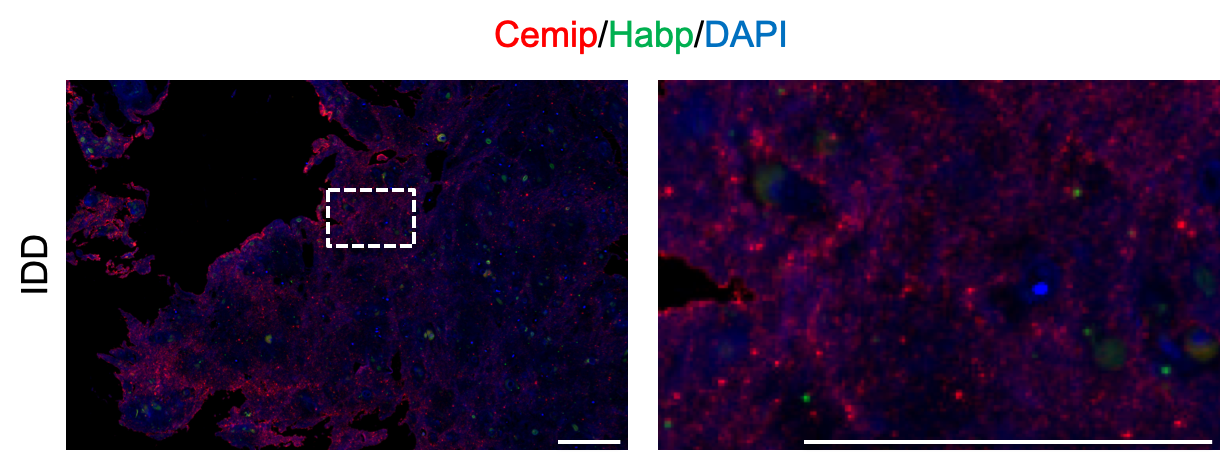


**Fig. S9.** Co-immunofluorescent staining of Cemip and Habp in degenerative human NP tissues. Scale bar, 200 μm.

Fig. S10.


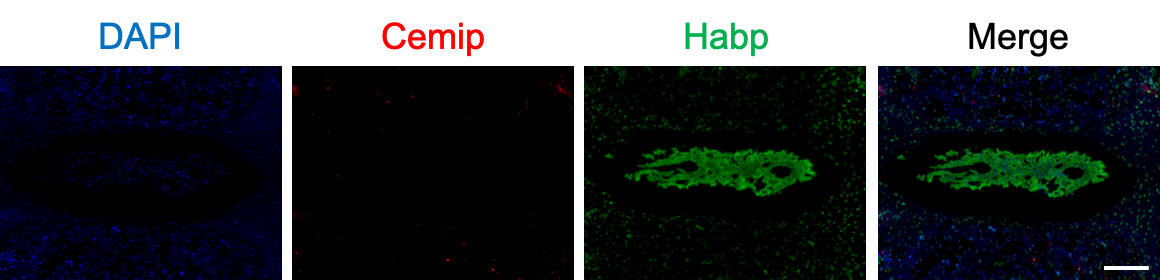


**Fig. S10.** Co-immunofluorescent staining of Cemip and Habp in the NP tissues of the KO mice. Scale bar, 200 μm.

Fig. S11.


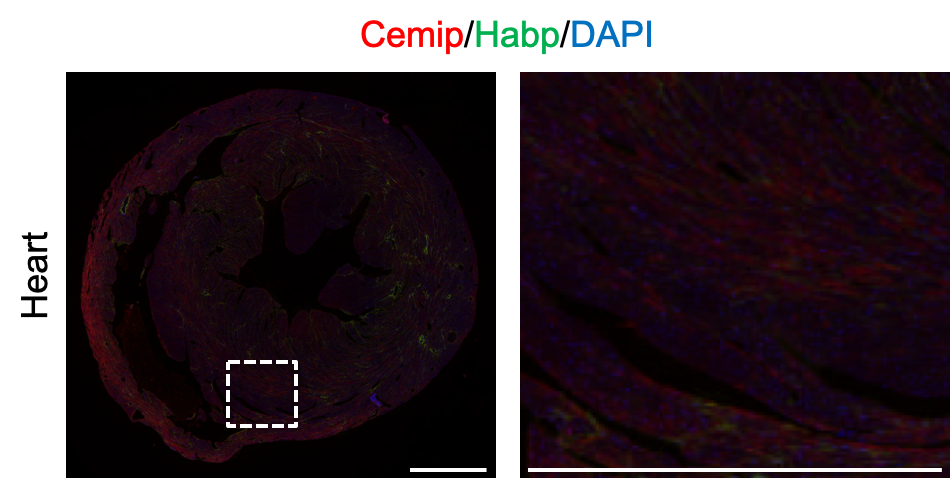


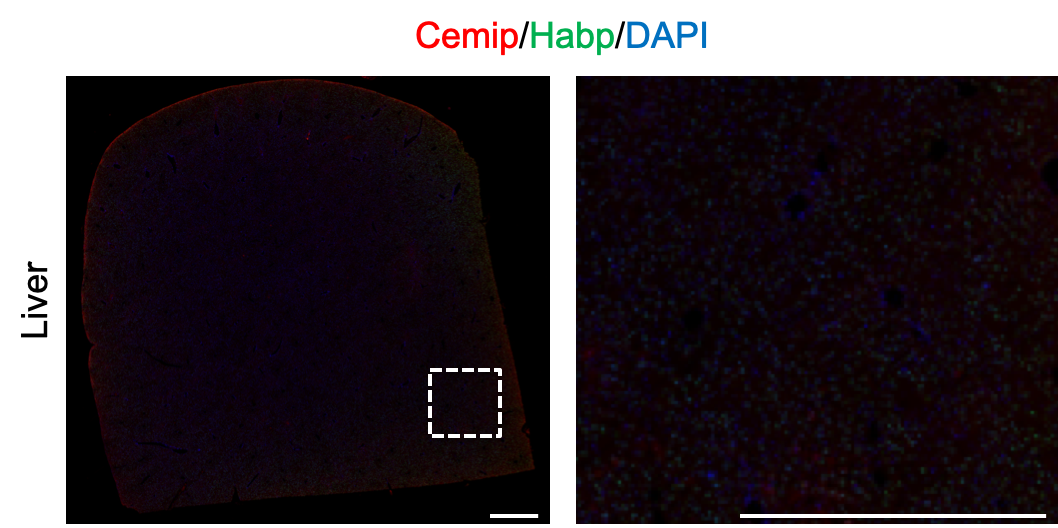


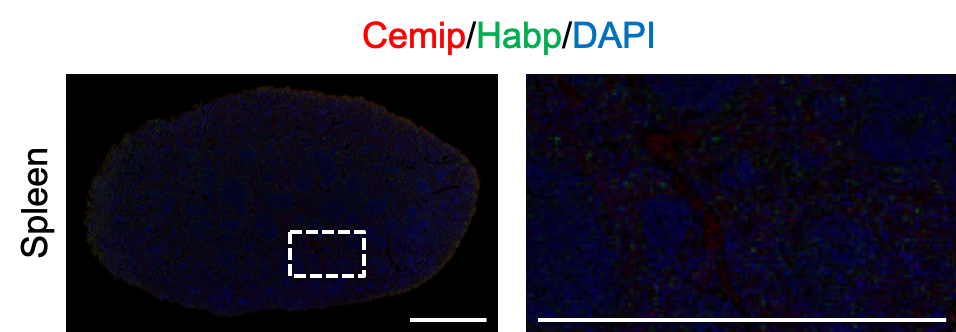


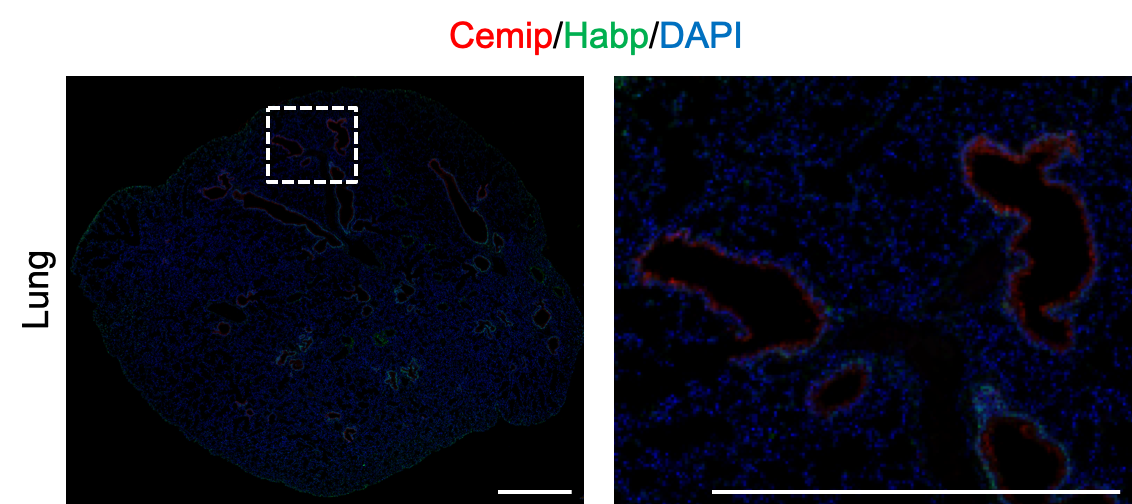


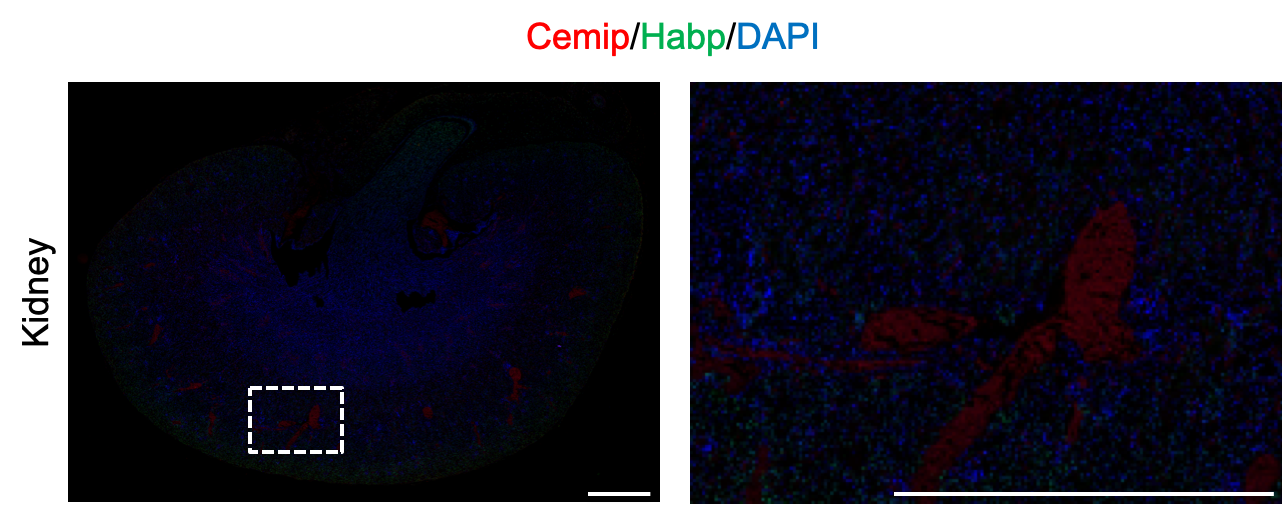


**Fig. S11.** Co-immunofluorescent staining of Cemip and Habp in liver, kidney, spleen, heart, and lungs of the KO mice. Scale bar, 1000 μm.

**Additional tables. (separate file)**

**TableS1_ ATAC_Up_Down_GO_Pathway**

**TableS2_RNA_LDND_GO_Pathway**

**TableS3_RNA_CEMIP_GO**

**TableS4_RNA_CEMIP_GSEA**

**TableS5_RNA_CEMIP_diffGene**

Table S6. **Characteristics details of the patients enrolled in the study.**

| **no.** | **Age** | **Gender** | **Diagnosis** | **Pfirrmann** |
| --- | --- | --- | --- | --- |
| **Case 1** | 76 | F | Lumbar spinal stenosis | V |
| **Case 2** | 56 | M | Lumbar disc herniation | V |
| **Case 3** | 57 | M | Lumbar disc herniation | V |
| **Case 4** | 72 | M | Lumbar spinal stenosis | V |
| **Case 5** | 49 | F | Lumbar disc herniation | IV |
| **Case 6** | 39 | F | Lumbar disc herniation | IV |
| **Case 7** | 55 | F | Lumbar disc herniation | IV |
| **Case 8** | 62 | M | Lumbar spinal stenosis | V |
| **Case 9** | 58 | F | Lumbar disc herniation | V |
| **Case 10** | 50 | F | Lumbar disc herniation | IV |
| **Case 11** | 61 | F | Lumbar spinal stenosis | IV |
| **Case 12** | 56 | M | Lumbar disc herniation | IV |
| **Case 13** | 48 | F | Lumbar disc herniation | IV |
| **Case 14** | 65 | M | Lumbar spinal stenosis | V |
| **Case 15** | 62 | F | Lumbar spinal stenosis | V |
| **Case 16** | 45 | M | thoracolumbar fracture | II |
| **Case 17** | 11 | F | scoliosis | I |
| **Case 18** | 17 | M | scoliosis | I |
| **Case 19** | 47 | F | thoracolumbar fracture | II |
| **Case 20** | 8 | F | scoliosis | I |
| **Case 21** | 35 | M | thoracolumbar fracture | II |
| **Case 22** | 42 | M | thoracolumbar fracture | II |
| **Case 23** | 10 | F | scoliosis | I |
| **Case 24** | 9 | F | scoliosis | I |
| **Case 25** | 52 | M | thoracolumbar fracture | II |
| **Case 26** | 13 | F | scoliosis | I |
| **Case 27** | 18 | M | scoliosis | I |
| **Case 28** | 22 | F | scoliosis | I |
| **Case 29** | 35 | F | thoracolumbar fracture | II |
| **Case 30** | 15 | M | scoliosis | I |

**Table S7. siRNA sequences used in siRNA transfection**

| **siRNA** | **Sense (5’-3’)** | **Antisense (5’-3’)** |
| --- | --- | --- |
| CEMIP #1 | CCUAUAGAUCCAAGAAAGATT | UCUUUCUUGGAUCUAUAGGTT |
| CEMIP #2 | CACUACAAUGGAUGGAGUUTT | AACUCCAUCCAUUGUAGUGTT |
| CEMIP #3 | GGCAGGACUUCCUCUUCAATT | UUGAAGAGGAAGUCCUGCCTT |
| c-FOS #1 | GCAAGGUGGAACAGUUAUCTT | GAUAACUGUUCCACCUUGCTT |
| c-FOS #2 | AGGAGAAUCCGAAGGGAAATT | UUUCCCUUCGGAUUCUCCUTT |
| c-FOS #3 | AACCUGCUGAAGGAGAAGGAATT | UUCCUUCUCCUUCAGCAGGUUTT |
| Fra-1 #1 | GUGGAUGGUACAGCCUCAUUUTT | AAAUGAGGCUGUACCAUCCACTT |
| Fra-1 #2 | GGAAGGAACUGACCGACUUTT | AAGUCGGUCAGUUCCUUCCTT |
| Fra-1 #3 | GCCCAGAGACUUUGUAGAUTT | AUCUACAAAGUCUCUGGGCTT |
| Atf-3 #1 | GAUGAGAGAAACCUCUUUATT | UAAAGAGGUUUCUCUCAUCTT |
| Atf-3 #2 | AUGUCCUCUGCGCUGGAAUTT | AUUCCAGCGCAGAGGACAUTT |
| Atf-3 #3 | UCACAAAAGCCGAGGUAGCTT | GCUACCUCGGCUUUUGUGATT |

**Table S8. Antibody information.**

| Primary Antibody | Concentration | Supplier |
| --- | --- | --- |
| For Western blot | | |
| CEMIP | 1:500 | Santa Cruz Biotechnology, CA, USA |
| Col II | 1:500 | Proteintech, Wuhan, China |
| Col I | 1:500 | Boster, Wuhan, China |
| MMP13 | 1:1000 | Cell Signaling Technology,  Boston, USA |
| ADAMTS-5 | 1:500 | Abcam, Cambridge, UK |
| aggrecan | 1:1000 | Abcam |
| Atf-3 | 1:1000 | Cell Signaling Technology |
| Fra-1 | 1:1000 | Cell Signaling Technology |
| c-FOS | 1:1000 | Cell Signaling Technology |
| aSMA | 1:500 | Proteintech |
| GAPDH | 1:1000 | Cell Signaling Technology |
| For Immunofluorescent and immunohistochemical staining | | |
| CEMIP | 1:200 | Proteintech |
| Col II | 1:500 | Abcam |
| Col I | 1:200 | Boster |
| MMP13 | 1:300 | Abcam |
| MMP9 | 1:500 | Cell Signaling Technology |
| ADAMTS-5 | 1:200 | Abcam |
| aggrecan | 1:200 | Proteintech |
| aSMA | 1:300 | Proteintech |
| HABP | 1:200 | Proteintech |

**Table S9. The primer sequences used in RT-PCR analysis.**

| **Primers** |  | **Sequence (5’ to 3’)** |
| --- | --- | --- |
| CEMIP | F | AGATCGGGGCATGAAAATCCT |
|  | R | ATTCCACCCATCACTCGGTC |
| GAPDH | F | GAGAAGTATGACAACAGCCTCAA |
|  | R | GCCATCACGCCACAGTTT |
| MMP13 | F | ACTGAGAGGCTCCGAGAAATG |
|  | R | GAACCCCGCATCTTGGCTT |
| ADAMTS-5 | F | GAACATCGACCAACTCTACTCCG |
|  | R | CAATGCCCACCGAACCATCT |
| COL2A1 | F | TGGACGATCAGGCGAAACC |
|  | R | GCTGCGGATGCTCTCAATCT |
| aggrecan | F | ACTCTGGGTTTTCGTGACTCT |
|  | R | ACACTCAGCGAGTTGTCATGG |
| Atf-3 | F | CCTCTGCGCTGGAATCAGTC |
|  | R | TTCTTTCTCGTCGCCTCTTTTT |
| Fra-1 | F | CAGGCGGAGACTGACAAACTG |
|  | R | TCCTTCCGGGATTTTGCAGAT |
| c-FOS | F | GGGGCAAGGTGGAACAGTTAT |
|  | R | CCGCTTGGAGTGTATCAGTCA |
| c-JUN | F | TCCAAGTGCCGAAAAAGGAAG |
|  | R | CGAGTTCTGAGCTTTCAAGGT |
| COL1A1 | F | GAGGGCCAAGACGAAGACATC |
|  | R | CAGATCACGTCATCGCACAAC |
| aSMA | F | GAGCTACGAACTGCCTGAC |
|  | R | CTGTTATAGGTGGTTTCGTGGA |
